# Arylboronic Acid Pinacol Esters as Stable Boron Sources for Dihydrodibenzoborepin Derivatives and a Dibenzoborole

**DOI:** 10.3390/molecules29174024

**Published:** 2024-08-25

**Authors:** Himeko Kawaguchi, Kotomi Fuse, Nanoka Maeda, Takuya Kuwabara

**Affiliations:** Department of Chemistry and Biochemistry, Graduate School of Humanities and Sciences, Ochanomizu University, 2-1-1, Otsuka, Bunkyo-ku, Tokyo 112-8610, Japan

**Keywords:** 10,11-dihydrodibenzoborepin, dibenzoborole, dibenzoborepin, synthetic method, boronic acid pinacol ester

## Abstract

The general synthesis of boron-containing cyclic compounds (boracycles) necessitates toxic organotin precursors or highly reactive boron halides. Here, we report the synthesis of seven- and five-membered boracycles utilizing arylboronic acid pinacol esters (ArBpins) as stable boron sources. Grignard reagents generated from 2,2′-dibromodibenzyl or 2,2′-dibromobiphenyl reacted with ArBpins, where Ar = 9-anthryl (Anth), 2,4,6-trimethylphenyl (Mes), or 2,4,6-triisopropylphenyl (Tip), to give 10,11-dihydro-5*H*-dibenzo[*b*,*f*]borepins or dibenzoborole derivatives. This Bpin-based method was successfully applied to a one-shot double boracycle formation, providing a dihydrodibenzoborepin–anthracene–dihydrodibenzoborepin triad molecule in a good yield. The dihydrodibenzoborepin bearing the Anth group was directly converted to the unsaturated borepin by NBS/AIBN. All products were characterized by NMR, HRMS, and in some cases, single-crystal X-ray diffraction analysis. Additionally, the photophysical properties of the products are also reported.

## 1. Introduction

Boron-containing unsaturated cyclic compounds (hereafter referred to as boracycles) have been studied intensively in many fields, including materials science [1,2,3,4] and aromatic/antiaromatic chemistry [5,6]. This is due to their unique conjugation system involving the vacant p orbital of the boron atom. Of particular interest are fused boroles and borepins, five- and seven-membered boracycles, respectively, owing to their 4π-antiaromaticity and 6π-aromaticity as well as fluorescent properties [7,8,9,10].

**Scheme 1:**
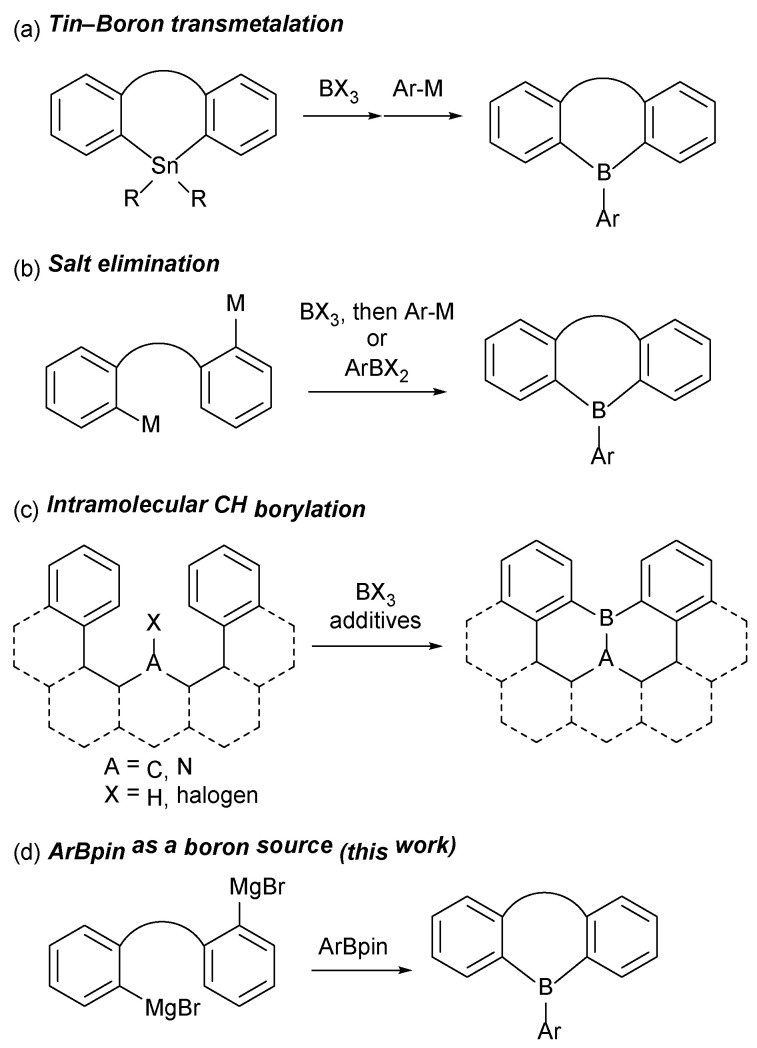
(**a**–**c**) General synthesis of boracycles and (**d**) ArBpin-based synthesis (this work). M = Li, MgBr; X = halogen.

Synthetic methods for boracycles can be roughly divided into three types: (1) tin–boron transmetalation, (2) salt-elimination reaction, and (3) intramolecular CH borylation (Scheme 1) [11,12,13,14]. The tin–boron transmetalation method has been a reliable route to many types of boracycles including dibenzoboroles (also known as 9-borafluorenes) and dibenzoborepins [8,15,16,17,18,19]. Similarly to the synthesis of other heterocycles [20], the salt-elimination reaction between metalated carbons, e.g., organolithiums and Grignard reagents, and haloboranes (BX_3_ and RBX_2_, where X = Cl, Br) is also widely employed [15]. Additionally, intramolecular cyclization via CH borylation has been developed as a convenient approach, particularly for the synthesis of boron-containing PAHs (polycyclic aromatic hydrocarbons) in the last decade [21,22,23,24]. However, the aforementioned methods rely on toxic organotin compounds and highly reactive haloboranes. Therefore, the development of alternative boracycle synthesis using less toxic chemicals is highly demanded from the viewpoint of sustainable chemistry.

One possible solution is using boronate esters [B(OR)_3_ and R′B(OR)_2_] as boron sources, and indeed, such boracycle formation has been reported. For example, the Yamaguchi’s group has reported the synthesis of dibenzoboroles and dibenzoborepins using ArB(OMe)_2_ as a boron source [10,25]. Additionally, stepwise boracycle formations involving arylboronic acid esters have also been developed [7,26,27,28]. Given the widespread application of arylboronic acid pinacol esters (ArBpins) in cross-coupling chemistry owing to their stability and moderate reactivity [29], we envisage that ArBpins can be useful and stable boron sources for boracycle synthesis. However, to the best of our knowledge, ArBpins have never been used as boron sources for boracycles, although the usage of B_2_pin_2_ for the synthesis of Mes_2_BBpin has been reported [30]. An additional advantage of using ArBpin is that its synthetic methods have been well-established: Miyaura borylation [31,32], CH borylation [33,34], and nucleophilic borylation. Therefore, the synthesis of boracycles using ArBpin would enable facile access to various boracycles which could serve as acceptor units in donor–acceptor-type molecules. In this report, we present the synthesis of seven- and five-membered boracycles utilizing ArBpin as the boron source. This method enables the straightforward synthesis of donor–acceptor- and acceptor–π–acceptor-type molecules with borepin acceptors. The photophysical properties of the newly synthesized borepin derivatives are also documented.

## 2. Results and Discussion

### 2.1. Synthetic Studies

The synthesis of 5-(9-anthryl)-5*H*-dibenzo[*b*,*f*]borepin **1-sp^2^** and its saturated analog **1-sp^3^** using AnthBpin (Anth = 9-anthryl) as a boron source was investigated (Table 1). The treatment of AnthBpin and dilithium reagents [(*Z*)-2,2′-dilithiostilbene (**2Li-sp^2^**) and 2,2′-dilithiodibenzyl (**2Li-sp^3^**)] did not provide **1-sp^2^** or **1-sp^3^** (entries 1 and 2). Switching the nucleophile to the Grignard reagent (**2Mg-sp^3^**) resulted in the isolation of **1-sp^3^** with a 62% yield (entry 3). Therefore, the type of nucleophile is important in this ArBpin-based boracycle synthesis. It is noteworthy that **1-sp^3^** was successfully purified by column chromatography, although it has a 9-anthryl group that is sterically less demanding compared to widely used protecting groups such as Mes (2,4,6-trimethylphenyl) and Tip (2,4,6-triisopropylphenyl). When AnthB(OMe)_2_ was employed as a boron source, the yield of **1-sp^3^** decreased to 31% (entry 4), indicating that the bidentate character of the pinacol group stabilizes a reaction intermediate and/or suppresses undesired side reactions. Our attempts to synthesize **1-sp^2^** by this method are unsuccessful because the preparation of Grignard reagent **2Mg-sp^2^** from (*Z*)-2,2′-dibromostilbene and magnesium causes the concomitant formation of phenanthrene and the *E*-isomer (entry 5 and Table S1). This type of *Z*/*E* isomerization of stilbene by alkaline metals and an Fe^I^ complex has been reported [35,36,37].

**Scheme 2:**
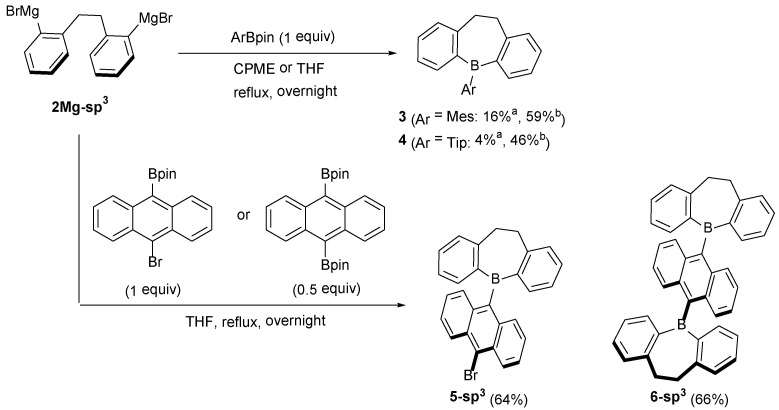
Reactions of **2Mg-sp^3^** and ArBpins. a: Yields in THF. b: Yields in CPME.

To explore the substrate scope of this ArBpin-based boracycle synthesis, other reagents were next subjected to the reaction (Scheme 2). When MesBpin [38] and TipBpin [39] were allowed to react with **2Mg-sp^3^** in THF under reflux, the yields of the corresponding dihydrodibenzoborepins **3** and **4** were only 16 and 4%, respectively. However, using cyclopentyl methyl ether (CPME) as a solvent improved the yields (59% for **3** and 46% for **4**). The higher boiling point of CPME (106 °C) compared to that of THF (66 °C) would be a key factor in the efficient reaction when bulky Ar groups are employed. The reaction using 9-Br-10-Bpin-anthracene [40] provided 10-bromoanthryl derivative **5-sp^3^** (64% yield), the bromo substituent of which can be used for further functionalization. Moreover, the one-shot double boracycle formation was achieved when 9,10-(Bpin)_2_-anthracene was used as a substrate to generate a dihydrodibenzoborepin–anthracene–dihydrodibenzoborepin triad molecule **6-sp^3^** in a 66% yield. On the contrary, our attempts to synthesize **6-sp^3^** using tin–boron transmetalation were unsuccessful; the reactions of in situ generated 5-chloro-5*H*-dibenzo[*b*,*f*]borepin, which was prepared from 5,5-dimethyl-5*H*-dibenzo[*b*,*f*]stannepin and BCl_3_, and 9,10-dilithioanthracene provided a complex mixture, highlighting the usefulness of the Bpin-based method.

**Scheme 3:**
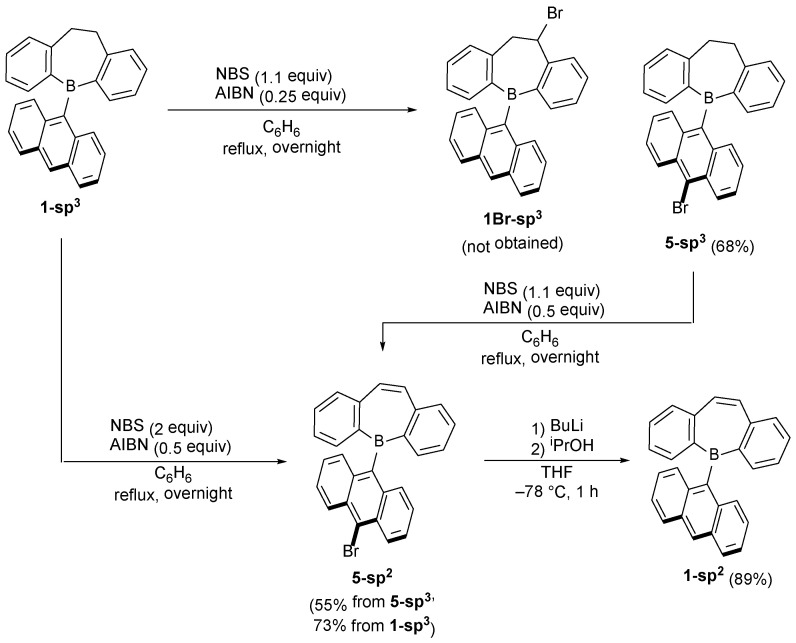
Reactions of saturated borepins and NBS/AIBN, and synthesis of **1-sp^2^**.

We next investigated the conversion of **1-sp^3^** to the corresponding unsaturated one (**1-sp^2^**) because the direct synthesis of **1-sp^2^** from the reaction in Table 1 failed (Scheme 3). According to the literature, the bromination of a dihydroborepin with *N*-bromosuccinimide (NBS) and subsequent elimination reaction using a base would be a promising route [41]. However, the bromination of **1-sp^3^** using 1.1 equiv NBS and 0.25 equiv azobis(isobutyronitrile) (AIBN) did not yield the benzylic bromination product **1Br-sp^3^** but **5-sp^3^** in a 68% yield. Thus, the bromination of **5-sp^3^** under the same conditions was next carried out, which provided a 3:2 mixture of **5-sp^3^** and **5-sp^2^**, and again, no **1Br-sp^3^** was obtained. Increasing the amount of AIBN to 0.5 equiv allowed the isolation of **5-sp^2^** with a 55% yield. Notably, one-pot synthesis of **5-sp^2^** was also achieved by a treatment of **1-sp^3^** with 2 equiv of NBS and 0.5 equiv of AIBN (73% yield). The bromo substituent in **5-sp^2^** was replaced by a hydrogen atom via a lithiation/protonation process to afford **1-sp^2^** in a 89% yield.

**Scheme 4:**
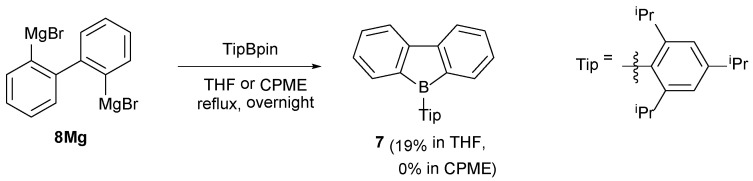
Synthesis of dibenzoborole **7** using the ArBpin-based method.

This ArBpin-based method is also applied for the synthesis of a dibenzoborole. We selected a Tip borole **7** as the target molecule because this molecule can be purified by column chromatography owing to the bulky Tip group [25]. Grignard reagent **8Mg** [42] generated from 2,2′-dibromobiphenyl reacted with TipBpin in THF under reflux to afford **7** in a 19% yield (Scheme 4). Although this yield is inferior to the reported method using **8Mg** and TipB(OMe)_2_ (45%) [25], it is worth noting that TipBpin can also be used as a stable boron source for dibenzoborole synthesis. In this case, the reaction in CPME instead of THF under reflux did not give **7** at all, probably due to the lower thermal stability of antiaromatic **7**.

### 2.2. Single-Crystal X-ray Diffraction Analysis

**Figure 1:**
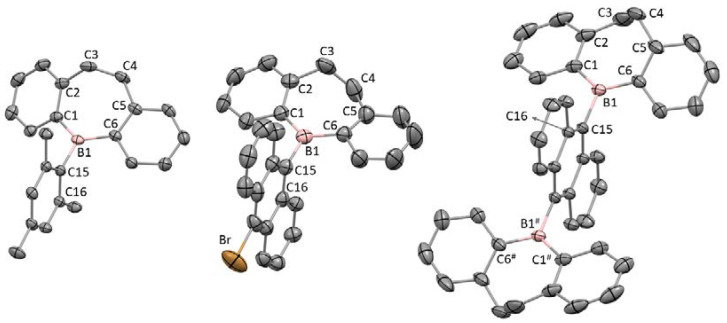
The crystal structures of **3** (**left**), **5-sp^3^** (**center**) and **6-sp^3^** (**right**) with thermal ellipsoid plots of a 50% probability. All hydrogen atoms and a co-crystalized chlorobenzene molecule in **6-sp^3^** are omitted for clarity. Only one of the two independent molecules are shown for **3** and **6-sp^3^**. Only the major disordered structure of **5-sp^3^** is shown.

Figure 1 illustrates the crystal structures of **3**, **5-sp^3^** and **6-sp^3^** and that of **4** is shown in Figure S2. Each asymmetric unit of **3**, **4** and **6-sp^3^** contains two independent molecules which are structurally similar. The dihydrodibenzoborepin skeleton of **5-sp^3^** is partially disordered over two positions with a ratio of 58:42. In the packing structure of **5-sp^3^**, the intermolecular Br…Br distance is 3.321 Å, much shorter than the sum of the van der Waals radii (3.70 Å), as shown in Figure S3, suggesting the existence of the halogen bond categorized as type I [43,44]. The packing structure of **6-sp^3^** shows that the CH-π and halogen-π interactions between the co-crystallized chlorobenzene molecule and the anthryl units of the two independent molecules are important (Figure S3) [45].

Table 2 shows the selected bond lengths and angles for these dihydrodibenzoborepins. Each boron atom has a planar three-coordinated structure with the sum of the C–B–C angles about 360°. These compounds have highly twisted structures with the C1–B1–C15–C16 torsion angles (80.2(2)–89.80(14)°) being larger than those in related 9-diarylborylanthracenes and 9,10-bis(diarylboryl)anthracenes (ca. 45–63°), where Ar = Mes and 2,6-dimethyl-4-trimethylammoniumphenyl [46,47]. Therefore, the bridging ethylene units in the dihydrodibenzoborepin skeletons induce a large dihedral angle between the dihydrodibenzoborepins and π- or donor units, which hinders effective conjugation between the vacant p orbital of the boron atom and the π electrons of the Mes and 9-anthryl groups. Accordingly, the B1–C15 bonds are slightly longer than the B1–C1/C6 bonds (ca. 1.59 vs. 1.57 Å). The dihydrodibenzoborepin skeletons are distorted, with the C1–C2–C5–C6 torsion angles around 20°, in contrast to the only slightly bent structures of aromatic dibenzoborepins [8,48].

### 2.3. Photophysical Properties and Theoretical Calculations

**Figure 2:**
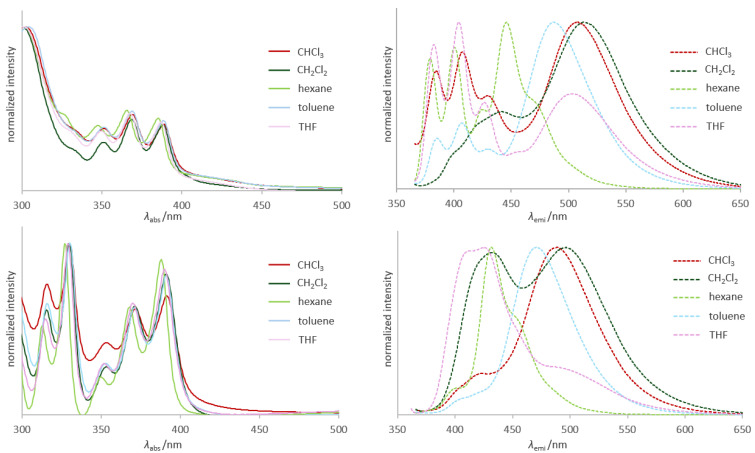
UV–vis absorption and emission spectra of **1-sp^3^** (**top**) and **1-sp^2^** (**bottom**) in various solvents (excited at 350 nm. *c* = 2.8 × 10^−5^ M). Solid lines indicate absorption spectra, and broken lines emission spectra.

Although the photophysical properties of aromatic dibenzoborepins are well-investigated [8,49], little is known about those of dihydrodibenzoborepins. Therefore, we explored the absorption and emission properties of **1-sp^3^** and **6-sp^3^** as well as **1-sp^2^**. Figure 2 shows the absorption and emission spectra of **1-sp^3^** and **1-sp^2^** recorded in different solvents (hexane, toluene, THF, CHCl_3_ and CH_2_Cl_2_). Spectra for **6-sp^3^** were recorded only in CH_2_Cl_2_ due to the poor solubility in non-halogenated solvents (Figure S4). These photophysical data are summarized in Table 3.

No significant solvent dependency was observed in the absorption spectra of **1-sp^3^** and **1-sp^2^**. Dihydrodibenzoborepin derivatives **1-sp^3^** and **6-sp^3^** show a broad and weak absorption ranging from ca. 450 to 400 nm, resulting from charge transfer (CT) from the anthracene unit to the dihydrodibenzoborepin acceptor, whereas the CT absorption is weak in **1-sp^2^** probably due to the aromatic character of borepin, which reduces the Lewis acidity of the boron atom [50]. Three absorption peaks of around 350–400 nm originating from the anthracene unit are slightly redshifted compared to those of anthracene by ca. 10 nm [51], the reason for which would be the inductive effect of the electropositive boron atoms (vide infra).

As mentioned above, the dihydrodibenzoborepin units are almost perpendicular to the central anthracene core, which causes a less effective conjugation between them. Therefore, it is important to compare the photophysical properties of **1-sp^3^**, **1-sp^2^**, **6-sp^3^** and related borylanthracenes. The absorption maxima of 9-[B(Mes)_2_]anthracene and 9,10-bis[B(Mes)_2_]anthracene, whose anthracene unit tilts ca. 53° with respect to the boron-centered plane, are 420 and 455 nm [52,53], redshifted compared to those of **1-sp^3^** and **6-sp^3^** (390 and 398 nm). This difference is rationalized by the different degree of π(anthracene)–p*(boron) conjugation.

In contrast to the absorption spectra, the emission spectra of **1-sp^3^** and **1-sp^2^** are highly dependent on the solvents, as commonly found in donor–acceptor-type molecules [54,55,56]. Stokes shifts of **1-sp^3^** varied from ca. 3500 to 6300 cm^−1^, being larger than those of **1-sp^2^**, as the solvent polarity increases. These larger Stokes shifts in **1-sp^3^** suggests that the dihydrodibenzoborepin skeleton is more flexible than the dibenzoborepin, allowing a greater structural relaxation in the excited state. Compound **1-sp^3^** shows two types of emission peaks: (1) an anthracene-based emission with three peak tops around 360 to 430 nm and (2) a broadened CT emission peak ranging from 450 to 530 nm. Interestingly, the strength of these two emissions depends on the solvent; the anthracene-based emission is dominant in THF, whereas the CT emission is a major contributor in the other solvents. The lower contribution of the CT emission in THF can be rationalized by the coordination of THF to the vacant p orbital of the boron atoms. Although a similar trend is found in the spectra of **1-sp^2^**, the anthracene-based emission peaks are broadened, the reason for which is not clear at this point.

**Figure 3:**
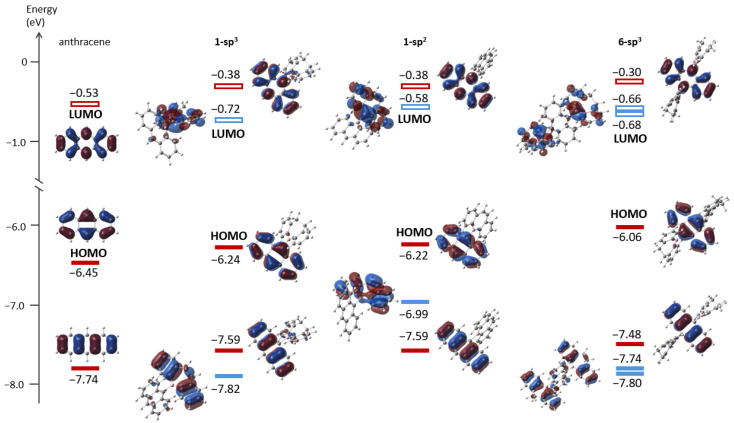
Frontier molecular orbitals and their energy levels (eV) for anthracene, **1-sp^3^**, **1-sp^2^** and **6-sp^3^** at CAM-B3LYP/6-31G(d,p)//B3LYP/6-31G(d) (isovalue = 0.03). Red bars: MOs mainly from anthracene. Blue bars: MOs mainly from (dihydro)dibenzoborepins.

To better understand the electronic structures, frontier molecular orbitals (MOs) for **1-sp^3^**, **1-sp^2^** and **6-sp^3^** as well as anthracene were calculated with CAM-B3LYP/6-31G(d,p)//B3LYP/6-31G(d) (Figure 3) [57,58]. As expected from the highly twisted structures, a π(anthracene)–p*(boron) conjugation is not found in the MOs. Importantly, the energy levels for the anthracene-based MOs increase as the number of the borepin unit increases, illustrated by the values of −6.45 eV (anthracene), −6.2 eV (**1-sp^3^** and **1-sp^2^**) and −6.06 eV (**6-sp^3^**) in the HOMOs, this due to the σ-donating character of the boron atoms [59]. The effective cyclic conjugation in the dibenzoborepin markedly raises the energy levels of the MOs derived from the dibenzoborepin moiety in **1-sp^2^** compared to those of the corresponding MOs in **1-sp^3^** [−6.99 eV (HOMO−1 in **1-sp^2^**) vs. −7.82 eV (HOMO−2 in **1-sp^3^**) and −0.58 eV (LUMO in **1-sp^2^**) vs. −0.72 eV (LUMO in **1-sp^3^**)]. These differences account for the strong absorption around 315 and 330 nm found only in **1-sp^2^**.

## 3. Materials and Method

### 3.1. General Considerations

All manipulations were performed under an argon atmosphere by using standard Schlenk techniques. Et_2_O, THF, hexane, benzene, toluene and CPME were dehydrated by 4A molecular sieves. All reagents were purchased from Sigma-Aldrich Japan (Tokyo, Japan), FUJIFILM Wako Pure Chemical Corporation (Osaka, Japan), Tokyo Chemical Industry Co., LTD. (Tokyo, Japan), Kanto Chemical Co., Inc. (Tokyo, Japan) or Nacalai Tesque (Kyoto, Japan) and used as received unless otherwise stated. Column chromatography was carried out using Wakogel silica 60N (particle size: 40–100 μm). (*Z*)-2,2′-Dibromostilbene [8], 2,2′-dibromodibenzyl [60], 2-(9-anthryl)-4,4,5,5-tetramethyl-1,3,2-dioxaborolane (AnthBpin) [61], 9,10-Bis(4,4,5,5-tetramethyl-1,3,2-dioxaborolan-2-yl)anthracene [Anth(Bpin)_2_] [62], AnthB(OMe)_2_ [63], 2-(2,4,6-trimethylphenyl)-4,4,5,5-tetramethyl-1,3,2-dioxaborolane (MesBpin) [38] and 2-(2,4,6-triisopropylphenyl)-4,4,5,5-tetramethyl-1,3,2-dioxaborolane (TipBpin) [64] were synthesized according to the literature. ^1^H, ^13^C{^1^H} and ^11^B{^1^H} NMR spectra were recorded on a JEOL ECZL-500R spectrometer at 20 °C unless otherwise stated. Chemical shifts are reported in δ and referenced to residual ^1^H and ^13^C{^1^H} NMR signals of the deuterated solvents as internal standards or to the ^11^B NMR signal of BF_3_·Et_2_O in CDCl_3_ (δ 0) as an external standard. Multiplicities are abbreviated as singlet (s), doublet (d), triplet (t), quartet (q), septet (sept), multiplet (m) and broad (br). Labels for NMR assignments are shown in Figure S1. The HRMS data were obtained by a Bruker ultrafleXtreme using 9-nitroanthracene as a matrix. Diffraction data were collected on a Bruker APEX II (for **3** and **6-sp^3^**) or Bruker D8 QUEST (for **4** and **5-sp^3^**) with Mo Kα radiation (λ = 0.71075 Å) at –110 to −80 °C. The structures were solved by direct methods using SHELXS. The refinements were performed using SHELXL-2019/3 [65]. The positions of the non-hydrogen atoms were determined by SHELXT 2018/2 [66]. All non-hydrogen atoms were refined on *F*_o_^2^ anisotropically by full-matrix least-square techniques. All hydrogen atoms were placed at the calculated positions with fixed isotropic parameters. UV–vis absorption and emission spectra were recorded using JASCO V-650 and JASCO FP-6600 spectrometers. Theoretical calculations were performed using the Gaussian 16 program [67]. The optimized structures of **1-sp^3^** and **6-sp^3^** are in good agreement with the corresponding X-ray structures. All local minima were confirmed by vibrational frequency calculations showing zero imaginary frequencies.

### 3.2. Preparation of ***2Mg-sp^3^***

THF (5.0 mL) was added to a 25 mL Schlenk tube containing Mg turnings (109.4 mg, 4.500 mmol, 3.0 equiv) and 2,2′-dibromodibenzyl (510.0 mg, 1.500 mmol) at room temperature. The reaction mixture was refluxed for 2 h. After cooling to room temperature, insoluble materials were filtered off under argon to give a gray solution of **2Mg-sp^3^** in THF (about 0.3 M).

### 3.3. Synthesis of 5-(9-Anthryl)-5H-9,10-dihydrodibenzo[b,f]borepin (***1-sp^3^***) Using AnthBpin

AnthBpin (71.0 mg, 0.193 mmol, 1.0 equiv) in THF (3.0 mL) was added to a solution of **2Mg-sp^3^** (0.3 M in THF, 0.64 mL, 0.19 mmol). The reaction mixture was refluxed overnight, after which the solvent was removed in vacuo. The product was purified by column chromatography (hexane:CH_2_Cl_2_ = 9:1) to yield **1-sp^3^** (44.1 mg, 0.120 mmol, 62%) as a yellow solid. ^1^H NMR (500 MHz, CDCl_3_): δ = 8.47 (s, 1H, *o*), 8.05 (d, ^3^*J*_HH_ = 8.5 Hz, 2H, *m*), 7.46 (dd, ^3^*J*_HH_ = 8.5 Hz, ^4^*J*_HH_ = 1.0 Hz, 2H, *j*), 7.40 (ddd, ^3^*J*_HH_ = 8.5 Hz, ^3^*J*_HH_ = 6.5 Hz, ^4^*J*_HH_ = 1.0 Hz, 2H, *l*), 7.36 (td, ^3^*J*_HH_ = 7.5 Hz, ^4^*J*_HH_ = 1.5 Hz, 2H, *d*), 7.33 (dd, ^3^*J*_HH_ = 7.5 Hz, ^4^*J*_HH_ = 1.5 Hz, 2H, *c*), 7.29 (dd, ^3^*J*_HH_ = 7.5 Hz, ^4^*J*_HH_ = 1.5 Hz, 2H, *f*), 7.21 (ddd, ^3^*J*_HH_ = 8.5 Hz, ^3^*J*_HH_ = 6.5 Hz, ^4^*J*_HH_ = 1.0 Hz, 2H, *k*), 6.91 (td, ^3^*J*_HH_ = 7.5 Hz, ^4^*J*_HH_ = 1.5 Hz, 2H, *e*), 3.45 (s, 4H, *a*); ^13^C{^1^H} NMR (126 MHz, CDCl_3_): δ = 152.5 (4°, *b*), 146.3 (4°, *h*), 143.2 (3°, *f*), 140.1 (4°, *g*), 133.7 (3°, *d*), 133.3 (4°, *i*), 131.3 (4°, *n*), 130.3 (3°, *j*), 128.81 (4°, *c*), 128.78 (3°, *m*), 126.00 (3°, *e*), 125.95 (3°, *o*), 125.1 (3°, *l*), 124.6 (3°, *k*), 37.9 (2°, a); ^11^B{^1^H} NMR (160 MHz, CDCl_3_): δ = 71.1; HRMS *m*/*z* calcd for C_28_H_21_B^+^ [M]^+^: 368.1736, found: 368.1736; Mp: 142 °C (decomp.).

### 3.4. Synthesis of 5-(2,4,6-Trimethylphenyl)-5H-9,10-dihydrodibenzo[b,f]borepin (***3***)

THF (2.0 mL) was added to a two-neck flask containing granular Mg (0.0878 mg, 3.61 mmol). To this flask, a THF solution (2.0 mL) of 2,2′-dibromodibenzyl (0.4081 g, 1.200 mmol) was added dropwise. After refluxing for 25 h, the solution was transferred to a Schlenk flask by a syringe to remove the remaining Mg. A THF solution of thus obtained **2Mg-sp^3^** (estimated to be 0.3 M. 1.97 mL, ca. 0.59 mmol) was added to a two-neck flask containing MesBpin (0.1320 g, 0.5363 mmol) and 5.8 mL of CPME, and the mixture was refluxed for 33 h. After removing the solvent in vacuo, the crude product was purified by column chromatography (eluent: hexane:EtOAc = 20:1) followed by washing with ^i^PrOH to give **3** as a white powder (98.9 mg, 0.318 mmol, 59%). Using THF instead of CPME decreased the yield to 16%. Single crystals suitable for X-ray diffraction analysis were obtained by a CH_2_Cl_2_/^i^PrOH solution. ^1^H NMR (500 MHz, CDCl_3_) δ 7.59 (d, *^3^J*_HH_ = 7.5 Hz, 2H, *f*), 7.40 (t, ^3^*J*_HH_ = 7.5 Hz, 2H, *d*), 7.26 (d, ^3^*J*_HH_ = 7.5 Hz, 2H, *c*), 7.15 (t, *^3^J*_HH_ = 7.5 Hz, 2H, *d*), 6.87 (s, 2H, *j*), 3.25 (s, 4H, *a*), 2.38 (s, 3H, *m*), 1.97 (s, 6H, *l*). ^13^C{^1^H} NMR (126 MHz, CDCl_3_) δ 152.6 (4°, *b*), 145.3 (4°, *h*), 141.9 (3°, *f*), 139.2 (4°, *g*), 137.4 (4°, *i*), 136.4 (4°, *k*), 133.3 (3°, *d*), 128.7 (3°, *c*), 127.0 (3°, *j*), 126.0 (3°, *e*), 37.7 (2°, *a*), 22.7 (1°, *l*), 21.4 (1°, *m*). ^11^B{^1^H} NMR (160 MHz, CDCl_3_) δ 69.3. HRMS for C_23_H_23_B (3) [M]^+^: 310.1893. Found: 310.1881. M.P.: 143 °C.

### 3.5. Synthesis of 5-(2,4,6-Triisopropylphenyl)-5H-9,10-dihydrodibenzo[b,f]borepin (***4***)

A THF solution of **2Mg-sp^3^** (estimated to be 0.3 M. 1.30 mL, ca. 0.39 mmol) was added to a two-neck flask containing TipBpin (0.1287 g, 0.3900 mmol) and 5.5 mL of CPME, and the mixture was refluxed for 22 h. After removing the solvent in vacuo, the crude product was purified by column chromatography (eluent: hexane:CH_2_Cl_2_ = 5:1) followed by PTLC (eluent: hexane:CH_2_Cl_2_ = 3:1) to give **4** as a white powder (69.9 mg, 0.177 mmol, 45%). Using THF instead of CPME decreased the yield to 4%. Single crystals suitable for X-ray diffraction analysis were obtained by a CH_2_Cl_2_/^i^PrOH solution. ^1^H NMR (500 MHz, CDCl_3_) δ 7.65 (dd, ^3^*J*_HH_ = 7.5 Hz, ^4^*J*_HH_ = 1.5 Hz, 2H, *f*), 7.38 (td, ^3^*J*_HH_ = 7.5 Hz, ^4^*J*_HH_ = 1.5 Hz, 2H, *d*), 7.25 (d, ^3^*J*_HH_ = 7.5 Hz, 2H, *c*), 7.16 (t, ^3^*J*_HH_ = 7.5 Hz, ^4^*J*_HH_ = 1.5 Hz, 2H, *e*), 7.00 (s, 2H, *j*), 3.23 (s, 4H, *a*), 2.96 (sept, ^3^*J*_HH_ = 6.9 Hz, 1H, *n*), 2.27 (sept, ^3^*J*_HH_ = 6.7 Hz, 2H, *l*), 1.33 (d, ^3^*J*_HH_ = 6.9 Hz, 6H, *o*), 0.96 (d, ^3^*J*_HH_ = 6.7 Hz, 12H, *m*). ^13^C{^1^H} NMR (126 MHz, CDCl_3_) δ 152.1 (4°, *b*), 148.6 (4°, *i*), 148.1 (4°, *k*), 143.3 (3°, *f*), 142.6 (4°, *h*), 140.2 (4°, *g*), 133.1 (3°, *d*), 128.6 (3°, *c*), 125.4 (3°, *e*), 120.2 (3°, *j*), 37.8 (2°, *a*), 35.5 (3°, *l*), 34.3 (3°, *n*), 24.4 (1°, *m*), 24.1 (1°, *o*). ^11^B{^1^H} NMR (160 MHz, CDCl_3_) δ 68.9. HRMS for C_29_H_35_B (**1a**) [M+H]^+^: 395.2832, found: 395.2920. M.P.: 126 °C.

### 3.6. Synthesis of 5-(10-Bromo-9-anthryl)-5H-9,10-dihydrodibenzo[b,f]borepin (***5-sp^3^***)

A solution of **2Mg-sp^3^** (0.3 M in THF, 8.2 mL, 2.46 mmol) was added to a THF (30 mL) solution of BrAnthBpin (901.0 mg, 2.455 mmol, 1.0 equiv). The reaction mixture was refluxed overnight, after which the solvent was removed in vacuo. The product was purified by column chromatography (hexane:CH_2_Cl_2_ = 9:1) to yield **5-sp^3^** (698.7 mg, 1.571 mmol, 64%) as a yellow solid. Single crystals suitable for X-ray diffraction analysis were obtained by the slow diffusion of hexane into a CH_2_Cl_2_ solution. ^1^H NMR (500 MHz, CDCl_3_): δ = 8.59 (dt, ^3^*J*_HH_ = 9.0 Hz, ^4^*J*_HH_ = 1.0 Hz, 2H, *m*), 7.53 (ddd, ^3^*J*_HH_ = 9.0 Hz, ^3^*J*_HH_ = 6.5 Hz, ^4^*J*_HH_ = 1.0 Hz, 2H, *l*), 7.46 (dt, ^3^*J*_HH_ = 8.5 Hz, ^4^*J*_HH_ = 1.0 Hz, 2H, *j*), 7.37 (td, ^3^*J*_HH_ = 7.5 Hz, ^4^*J*_HH_ = 1.0 Hz, 2H, *d*), 7.34 (dd, ^3^*J*_HH_ = 7.5 Hz, ^4^*J*_HH_ = 1.5 Hz, 2H, *c*), 7.26 (d, 2H, *f*), 7.24 (m, 2H, *k*), 6.91 (td, ^3^*J*_HH_ = 7.5 Hz, ^4^*J*_HH_ = 1.5 Hz, 2H, *e*), 3.44 (s, 2H, *a*). ^13^C{^1^H} NMR (126 MHz, CDCl_3_): δ = 152.6 (4°, *b*), 147.0 (4°, *h*), 143.3 (3°, *f*), 139.7 (4°, *g*), 133.9 (3° and 4°, *d* and *i*), 130.7 (3°, *j*), 130.1 (4°, *n*), 128.9 (3°, *c*), 128.2 (3°, *m*), 126.9 (3°, *l*), 126.0 (3°, *e*), 124.8 (3°, *k*), 122.2 (4°, *o*), 37.8 (2°, *a*). ^11^B{^1^H} NMR (160 MHz, CDCl_3_): δ = 70.6. HRMS *m*/*z* calcd for C_28_H_20_BBr^+^ [M]^+^: 446.0841, found: 446.0898. Mp: 113 °C (decomp.).

### 3.7. Synthesis of 9,10-Bis(5H-9,10-Dihydrodibenzo[b,f]borepin-5-yl)anthracene (***6-sp^3^***)

A solution of **2Mg-sp^3^** (0.3 M in THF, 9.27 mL, 2.78 mmol, 2.0 equiv) was added to a solution of **Anth(Bpin)_2_** (598.6 mg, 1.392 mmol, 1.0 equiv) in THF (24.0 mL). The reaction mixture was refluxed overnight, during which a yellow solid precipitated. The yellow solid insoluble in THF was filtered off and washed with CH_2_Cl_2_ to yield **6-sp^3^** (414.7 mg, 0.9187 mmol, 66%) as a yellow solid. Single crystals suitable for X-ray diffraction analysis were obtained by standing a saturated chlorobenzene solution of **6-sp^3^**. ^1^H NMR (500 MHz, CDCl_3_): δ = 7.50 (dd, ^3^*J*_HH_ = 8.0 Hz, 4H, *j*), 7.48 (dd, ^3^*J*_HH_ = 7.5 Hz, ^4^*J*_HH_ = 1.5 Hz, 4H, *f*), 7.41 (td, ^3^*J*_HH_ = 7.5 Hz, ^4^*J*_HH_ = 1.5 Hz, 4H, *d*), 7.35 (td, ^3^*J*_HH_ = 8.0 Hz, ^4^*J*_HH_ = 1.5 Hz, 4H, *c*), 7.12 (dd, ^3^*J*_HH_ = 7.0 Hz, 4H, *k*), 7.02 (td, 4H, ^3^*J*_HH_ = 7.5 Hz, ^4^*J*_HH_ = 1.5 Hz, *e*), 3.48 (s, 8H, *a*); ^13^C{^1^H} NMR (126 MHz, CDCl_3_): δ = 152.4 (4°, *b*), 143.3 (3°, *f*), 133.7 (3°, *d*), 132.8 (4°, *i*), 130.8 (3°, *j*), 128.8 (3°, *c*), 126.1 (3°, *e*), 124.1 (3°, *k*), 37.9 (2°, *a*). Two signals (*g* and *h*) were not observed due to the low solubility in CDCl_3_ as well as the quadrupolar relaxation caused by ^11^B. ^11^B{^1^H} NMR signals could also not be observed due to low solubility. HRMS *m*/*z* calcd for C_42_H_32_B_2_^+^ [M]^+^: 558.2690, found: 558.2721. Mp: 151 °C (decomp.).

### 3.8. Synthesis of ***5-sp^2^*** from ***5-sp^3^***

NBS (88.9 mg, 0.499 mmol, 1.1 equiv) and AIBN (37.3 mg, 0.227 mmol, 50 mol%) were added to a solution of **5-sp^3^** (203.0 mg, 0.4540 mmol) in benzene (8.0 mL) at room temperature. The reaction mixture was refluxed overnight, after which the solvent was removed in vacuo. The product was purified by short pad column chromatography (hexane:CH_2_Cl_2_ = 5:3). The obtained yellow solid was dissolved in CH_2_Cl_2_ and cooled at −15 °C to yield **5-sp^2^** (112.0 mg, 55%) as a yellow solid. ^1^H NMR (500 MHz, CDCl_3_): δ = 8.65 (dt, ^3^*J*_HH_ = 9.0 Hz, 2H, *m*), 7.89 (dt, ^3^*J*_HH_ = 9.0 Hz, 2H, *c*), 7.70 (m, 4H, *d* + *j*), 7.54 (m, 4H, *a* + *l*), 7.37 (d, ^3^*J*_HH_ = 7.5 Hz, 2H, *f*), 7.18 (m, 4H, *e* + *k*). ^13^C{^1^H} NMR (126 MHz, CDCl_3_): δ = 147.5 (4°, *h*), 144.4 (4°, *b*), 142.8 (3°, *f*), 140.6 (4°, *g*), 134.5 (3° and 4°, *a* and *i*), 133.4 (3°, *d*), 133.2 (3°, *c*), 131.2 (3°, *j*), 130.2 (4°, *n*), 128.2 (3°, *m*), 127.4 (3°, *e*), 126.9 (3°, *l*), 124.8 (3°, *k*), 122.2 (4°, *o*). ^11^B{^1^H} NMR (160 MHz, CDCl_3_): δ = 65.9. HRMS *m*/*z* calcd for C_28_H_18_BBr^+^ [M]^+^: 444.0685, found: 444.0640. Mp: 169 °C (decomp.).

### 3.9. Synthesis of ***1-sp^2^*** from ***5-sp^2^***

*n*-BuLi (2.0 M in cyclohexane, 0.084 mL, 0.168 mmol, 1.1 equiv) was added to a solution of **5-sp^2^** (68.1 mg, 0.1529 mmol) in THF (1.0 mL) at −78 °C. After 1 h, the reaction mixture was quenched with ^i^PrOH. The solvents were evaporated at reduced pressure. Compound **1-sp**^2^ was obtained as yellow solid (49.9 mg, 0.136 mmol, 89%). ^1^H NMR (600 MHz, CDCl_3_): δ = 8.56 (s, 1H, *o*), 8.11 (d, ^3^*J*_HH_ = 8.5 Hz, 2H, *m*), 7.90 (dd, ^3^*J*_HH_ = 8.0 Hz, 2H, *c*), 7.75 (d, ^3^*J*_HH_ = 8.0 Hz, 2H, *f*), 7.70 (t, ^3^*J*_HH_ = 7.5 Hz, 2H, *d*), 7.54 (s, 2H, *a*), 7.42 (t, ^3^*J*_HH_ = 7.5 Hz, 2H, *l*), 7.38 (d, ^3^*J*_HH_ = 8.0 Hz, 2H, *j*), 7.18 (t, ^3^*J*_HH_ = 7.8 Hz, 2H, *e*), 7.14 (t, ^3^*J*_HH_ = 8.0 Hz, 2H, *k*). ^13^C{^1^H} NMR (150 MHz, CDCl_3_): δ = 146.7 (4°, *h*), 144.2 (4°, *b*), 142.8 (3°, *f*), 140.9 (4°, *g*), 134.5 (3°, *a*), 133.8 (4°, *i*), 133.2 (3°, *d*), 133.0 (3°, *c*), 131.3 (4°, *n*), 130.7 (3°, *j*), 128.8 (3°, *m*), 127.3 (3°, *e*), 125.9 (3°, *o*), 125.0 (3°, *l*), 124.5 (3°, *k*). ^11^B{^1^H} NMR (160 MHz, CDCl_3_): δ = 66.1. HRMS *m*/*z* calcd for C_28_H_19_B^+^ [M]^+^: 366.1580, found: 366.1564. Mp: 186 °C (decomp.).

### 3.10. Synthesis of ***7***

A two-necked flask was charged with 2,2′-dibromobiphenyl (177.1 mg, 0.5677 mmol), Mg (27.6 mg, 1.135 mmol) and THF (1.4 mL). After refluxing this mixture for 1 h, a THF solution of TipBpin (131.1 mg, 0.3973 mmol) was added to the flask at room temperature. The mixture was refluxed for 24 h to give yellow solution, the solvent of which was removed under reduced pressure. Crude mixture thus obtained was purified by column chromatography (eluent: hexane:CH_2_Cl_2_ = 20:1) to give **7** as a yellow powder (27.9 mg, 0.0762 mmol, 19% yield). The ^1^H NMR data of **7** were identical to those of the reported one in ref. [25].

## 4. Conclusions

We have developed a novel synthetic method for dihydrodibenzoborepin and dibenzoborole skeletons utilizing ArBpins as stable boron sources, which exclude the use of toxic organotin reagents and haloboranes. This method enabled the one-shot double formation of boracycles to give **6-sp^3^**, which could not be obtained by a well-established transmetalation route using an organotin compound. The dihydrodibenzoborepins **1-sp^3^** and **5-sp^3^** were directly converted into dibenzoborepin **5-sp^2^** under refluxing with NBS/AIBN. In the crystalline state, compounds **3**, **4**, **5-sp^3^** and **6-sp^3^** adopt a highly twisted structure because of the steric hindrance between the bulky Ar substituents and the dihydrodibenzoborepins. Spectroscopic studies reveal that **1-sp^3^** and **1-sp^2^** exhibit solvatofluorochromic properties. We believe that this Bpin-based boracycle synthesis would be a viable alternative to the conventional approach.

## Figures and Tables

**Table 1:** Synthesis of **1-sp^2^** and **1-sp^3^** using AnthBpin and AnthB(OR)_2_.

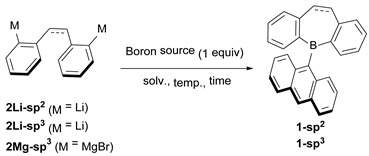				
Entry	Boron Source	Nucleophile	Conditions	Yield
1	AnthBpin	**2Li-sp^2^**	C_6_H_6_/Et_2_O, 5 °C to r.t., overnight	**1-sp^2^** (–)
2	AnthBpin	**2Li-sp^3^**	C_6_H_6_/Et_2_O, 5 °C to r.t., overnight	**1-sp^3^** (–)
3	AnthBpin	**2Mg-sp^3^**	THF, reflux, overnight	**1-sp^3^** (62%)
4	AnthB(OMe)_2_	**2Mg-sp^3^**	THF, reflux, overnight	**1-sp^3^** (31%)
5	AnthBpin	**2Mg-sp^2^** ^*a*^	THF, reflux, overnight	**1-sp^2^** (–)

*^a^* A mixture of **2Mg-sp^2^**, its *E*-isomer and phenanthrene was used.

**Table 2:** Selected bond lengths [Å] and angles [°].

	3	5-sp^3^	6-sp^3^
B1–C1/C6	1.5682(18)/ 1.5706(19)	1.562(5)/ 1.567(5)	1.572(3)/ 1.569(4)
B1–C15	1.5866(17)	1.592(5)	1.594(3)
C1–B1–C15–C16	89.80(14)	89.2(4)	80.2(2)
C1–C2–C5–C6	19.1(1)	20.2(6)	18.9(2)

**Table 3:** Absorption and emission properties of **1-sp^3^**, **1-sp^2^** and **6-sp^3^**.

	Solvent	λ_abs_/nm (ε)	λ_emi_/nm	Stokes Shift/cm^−1^
**1-sp^3^**	hexane	385 (9300), 366 (10,000), 348 (8300)	380, 400, 425, 446	3550
	toluene	388 (9300), 369 (11,000), 351 (8400)	385, 407, 431, 487	5240
	THF	387 (9700), 367 (11,000), 350 (8400)	382, 404, 426, 503	5960
	CHCl_3_	389 (7200), 369 (8300), 348 (6800)	384, 407, 430, 508	6020
	CH_2_Cl_2_	388 (9400), 369 (10,000), 351 (6900)	441, 513	6280
**1-sp^2^**	hexane	388 (14000), 368 (10,000), 350 (3900), 327 (16,000), 313 (8600)	432, 451	3600
	toluene	391 (17,000), 371 (13,000), 353 (6400), 330 (21,000), 316 (14,000)	471	4340
	THF	390 (15,000), 370 (12,000), 352 (5400), 329 (18,000), 315 (10,000)	410, 425	2110
	CHCl_3_	391 (15,000), 372 (13,000), 353 (9100), 330 (22,000), 316 (16,000)	489	5130
	CH_2_Cl_2_	391 (12,000), 371 (9200), 353 (4000), 330 (14,000), 316 (8700)	432, 496	5410
**6-sp^3^**	CH_2_Cl_2_	398 (9300), 378 (10,000), 360 (7900)	532	6330

## Data Availability

Crystallographic data for the reported compounds in this article have been deposited at the Cambridge Crystallographic Data Centre: CCDC 2371388 (**3**), 2371389 (**4**), 2371390 (**5-sp^3^**), 2371391 (**6-sp^3^**). These data can be obtained free of charge via https://www.ccdc.cam.ac.uk/structures/.

## Supplementary Materials

The following supporting information can be downloaded at https://www.mdpi.com/article/10.3390/molecules29174024/s1, Figure S1: labels for NMR assignments of the products; additional synthetic procedures; Table S1: preparation and quenching reactions of **2Mg-sp^2^**; Figure S2: molecular structures of **4**; Figure S3: packing structures of **5-sp^3^** and **6-sp^3^**; Figure S4: absorption (solid line) and emission spectra (broken line) of **6-sp^3^**; Table S2. crystallographic data for **3**, **4**, **5-sp^3^** and **6-sp^3^**; Cartesian coordinates for **1-sp^3^**, **1-sp^2^** and **6-sp^3^**; Figures S5–S24: ^1^H, ^13^C{^1^H} and ^11^B{^1^H} NMR spectra of the new compounds.
