# Supplementary material for: Arylboronic Acid Pinacol Esters as Stable Boron Sources for Dihydrodibenzoborepin Derivatives and a Dibenzoborole
Source: Molecules. 2024 Aug 25;29(17):4024. doi: 10.3390/molecules29174024 (PMC11397486; doi:10.3390/molecules29174024)
Supplement: Supplementary file 1 [file molecules-29-04024-s001.zip › molecules-3144990-supplementary.pdf]

Supporting information for

Arylboronic Acid Pinacol Esters as Stable Boron Sources for Dihydrodibenzoborepin Derivatives and a Dibenzoborole

Himeko Kawaguchi, Kotomi Fuse, Nanoka Maeda and Takuya Kuwabara *
Department of Chemistry and Biochemistry, Graduate School of Humanities and Sciences, Ochanomizu University, 2-1-1, Otsuka, Bunkyo-ku, Tokyo 112-8610, Japan; sxdcfv7u8i@gmail.com (H.K.); roadaheadlsp-1@yahoo.co.jp (K.F.); g2340640@edu.cc.ocha.ac.jp (N.M.)
* Correspondence: kuwabara.takuya@ocha.ac.jp

Table of contents

1. Experimental Details S1–2
2. Preparation of **2Mg-sp^2** and its quenching reactions S3
3. Molecular structure of **4** S4
4. Packing structures of **5-sp^3** and **6-sp^3** S5
5. Absorption and emission spectra of **6-sp^3** S6
6. Crystallographic data for **3**, **4**, **5-sp^3** and **6-sp^3** S7
7. Cartesian coordinates for **1-sp^3**, **1-sp^2** and **6-sp^3** S8–12
8. NMR spectra of the products S13–22

1. Experimental Details

Labels for NMR assignments

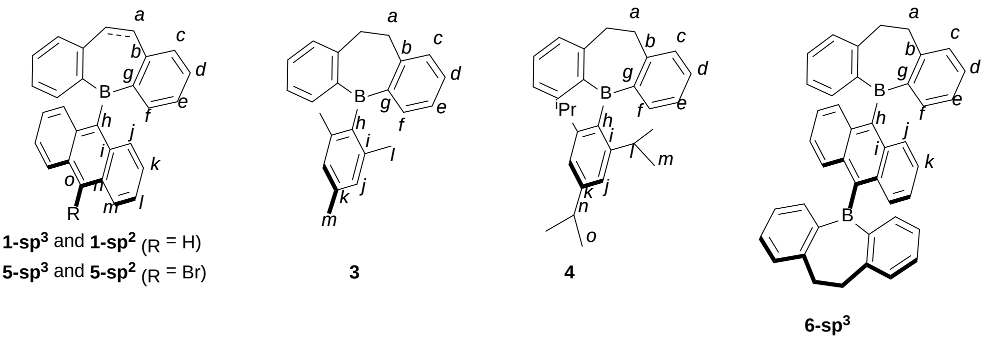

Figure S1. Labels for NMR assignments of the products.

Synthesis of 1-sp^3 using AnthB(OMe)$_2$

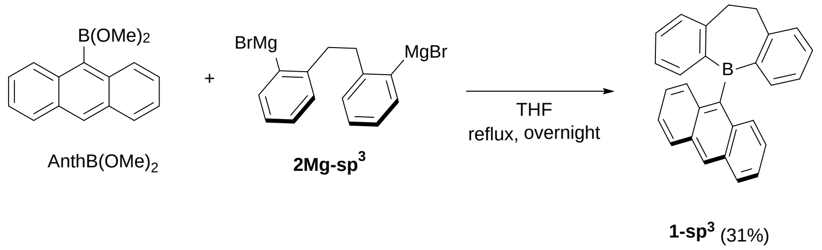

AnthB(OMe)$_2$ (59.1 mg, 0.237 mmol, 1.0 equiv) in THF (1.0 mL) was added to a solution of **2Mg-sp^3** (0.3 M in THF, 0.80 mL, 0.24 mmol) in THF (3.0 mL). The reaction mixture was refluxed overnight, after which the solvent was removed in vacuo. The product was purified by column chromatography (hexane:CH_2Cl_2 = 9:1) to yield **1-sp^3** (25.9 mg, 0.0735 mmol, 31 %) as a yellow solid.

Improved synthesis of 2-(10-bromo-9-anthryl)-4,4,5,5-tetramethyl-1,3,2-dioxaborolane (BrAnthBpin)

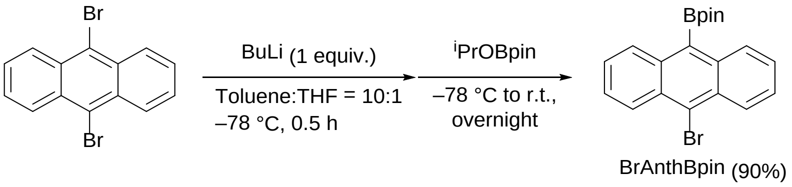

n-BuLi (2.0 M in cyclohexane, 1.50 mL, 3.00 mmol, 1.0 equiv) was added to a solution of 9,10-dibromoanthracene (1009.1 mg, 3.003 mmol) in toluene (100 mL) and THF (10 mL) at −78 °C. After 30 min, 2-isopropoxy-4,4,5,5-tetramethyl-1,3,2-dioxaboralane (0.74 mL, 3.60 mmol, 1.2 equiv) was added to the mixture, which was allowed to gradually warm to room temperature overnight and quenched with water. Organic phase was extracted by Et_2O (15 mL) twice and dried over $MgSO_4$. After filtration, the solvents were evaporated under reduced pressure. The crude product was purified by column chromatography (from hexane to hexane:AcOEt = 9:1) to yield BrAnthBpin (992.1 mg, 2.70 mmol, 90%) as a yellow solid. The ^{1}H NMR data corresponds to the reported one.[40]

Synthesis of 5-sp³ from 1-sp³

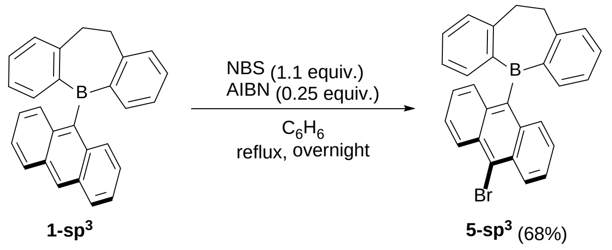

NBS (26.8 mg, 0.151 mmol, 1.1 equiv) and AIBN (5.6 mg, 0.034 mmol, 0.25 equiv) were added to a solution of **1-sp³** (50.1 mg, 0.137 mmol) in benzene (2.0 mL) at room temperature. The reaction mixture was refluxed overnight, after which the solvent was removed in vacuo. The product was purified by short pad column chromatography (hexane:CH_2Cl_2 = 9:1) to provide **5-sp³** in 68% yield.

Synthesis of 5-sp² from 1-sp³

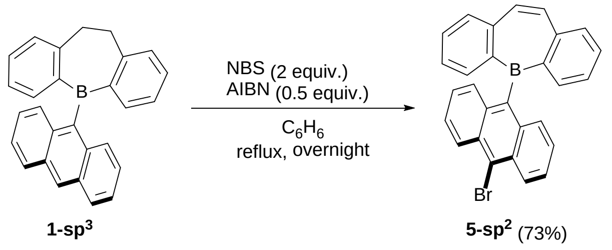

1-sp³ (198.7 mg, 0.5395 mmol) was added to a two-necked round-bottom flask and dissolved in 8 mL of benzene. To this solution, NBS (192.0 mg, 1.079 mmol) and AIBN (44.3 mg, 0.268 mmol) were added, and the mixture was refluxed overnight. After cooling to room temperature, the solvent was removed in vacuo. The crude product was purified by short pad column chromatography (eluent: hexane:CH_2Cl_2 = 5:1) to yield **5-sp²** (0.1759 g, 73% yield).

2. Preparation of 2Mg-sp^2 and its quenching reactions

During our attempts to synthesize **1-sp^2** from the reaction of AnthBpin and **2Mg-sp^2**, we found that phenanthrene and *E*-stilbene were also formed. This result prompted us to investigate the optimization of the reaction conditions for the formation of **2Mg-sp^2**. Table S1 summarizes the results of the reaction of (*Z*)-2,2′-dibromostilbene and magnesium under various conditions followed by quenching with H_2O. Unfortunately, none of the conditions gave satisfactory results. A synthetic attempt of **1-sp^2** using a mixture of **2Mg-sp^3** obtained by entry 2 failed (Table 1, entry 5).

Table S1. Preparation and quenching reactions of **2Mg-sp^2**.

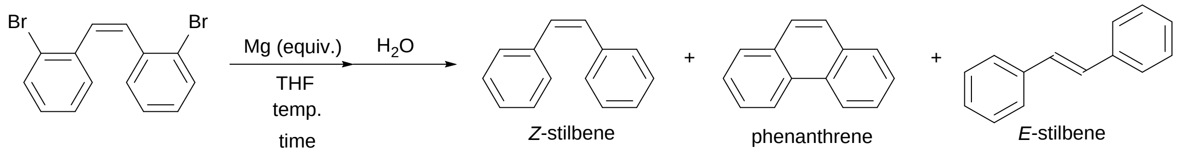

entry	Equiv of Mg	Temp.	Time (h)	*Z*-stilbene:phenanthrene:*E*-stilbene[a]
1	2	r.t.	1	6:1:3
2	3	r.t.	2	6:1:1
3	3	r.t.	14	3:2:1
4	3	reflux	1	3:2:1
5	3	reflux	14	0:1:6

[a]Estimated by ^{1}H NMR spectra of the crude products.

3. Molecular structure of 4

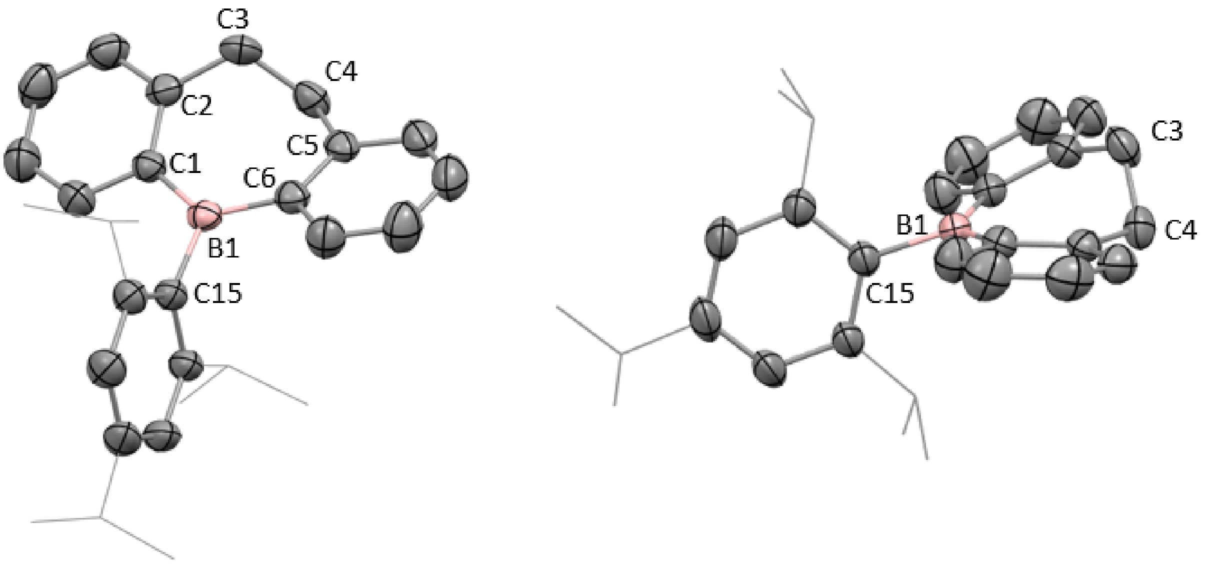

Figure S2. Molecular structures of **4** with thermal ellipsoid plots at 50% probability as well as the ^{i}Pr groups with wireframe. Only one of the two independent molecules is shown. All hydrogen atoms and a minor part of the disordered *p*-^{i}Pr group are omitted for clarity.

4. Packing structures of 5-sp^3 and 6-sp^3

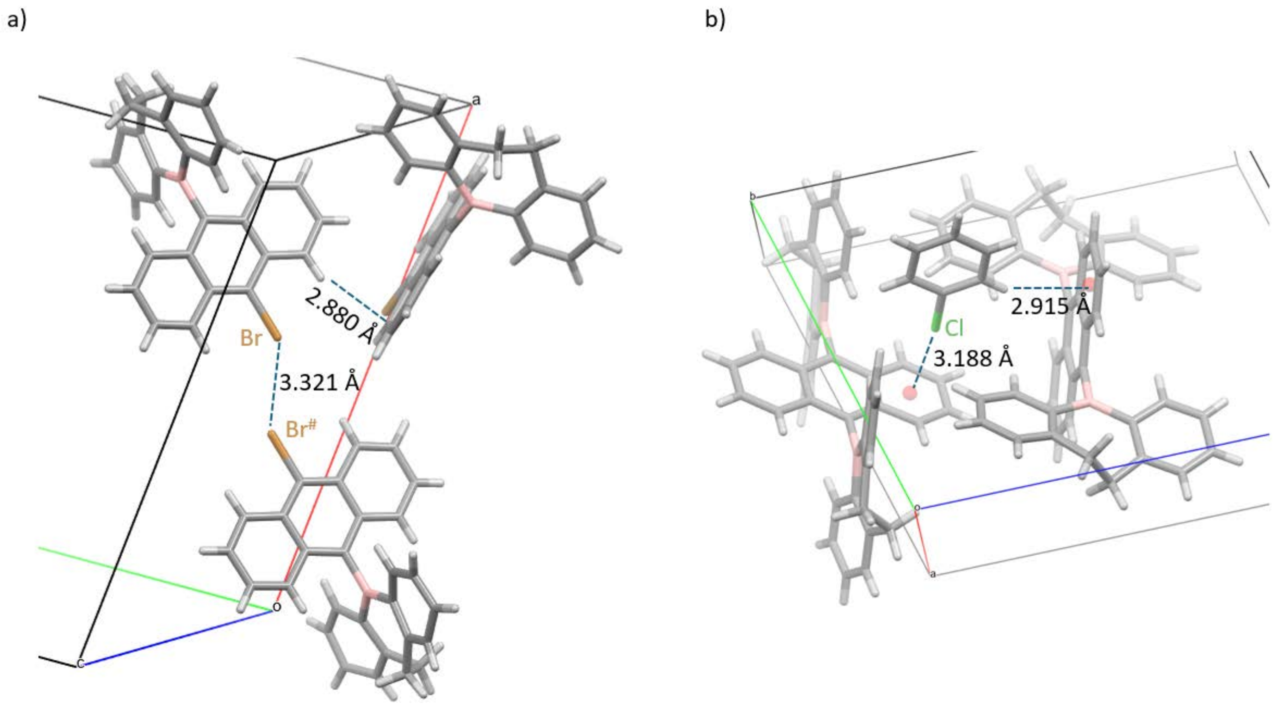

Figure S3. Packing structures of a) **5-sp^3** and b) **6-sp^3**. The minor part of the disordered dihydrodibenzoborepin in **5-sp^3** is not shown. Selected distances [Å] and angles [deg]: Br···Br: 3.321; CH···C_{anth}: 2.880; ∠C–Br···Br: ·145.3 for **5-sp^3**. Cl···center the C_6 ring: 3.188; CH···center of the C_6 ring: 2.915; ∠C–Cl···center the C_6 ring: 172.5 for **6-sp^3**.

5. Absorption and emission spectra of 6-sp^3

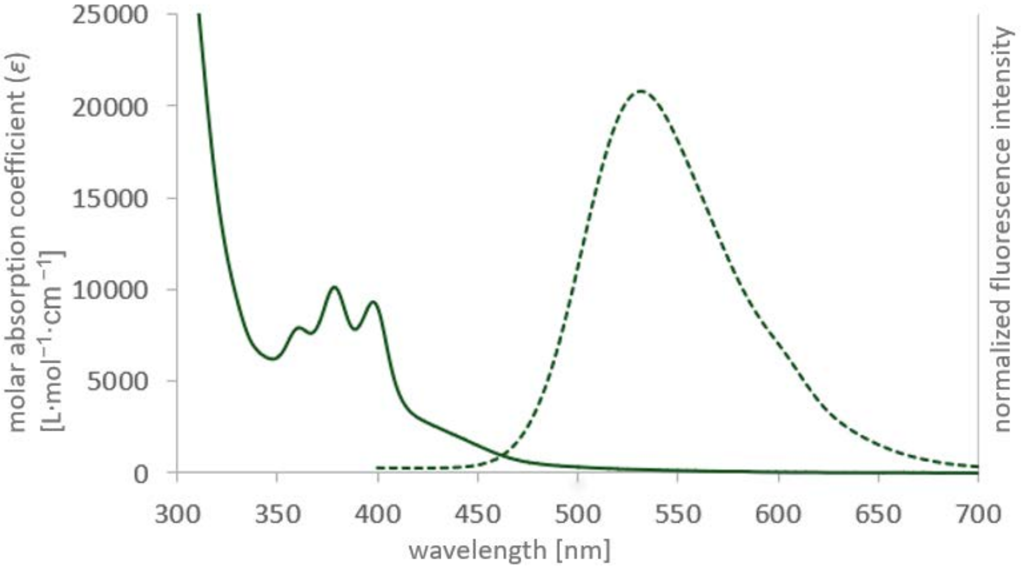

Figure S4. Absorption (solid line) and emission spectra (broken line) of **6-sp^3** recorded in CH_2Cl_2 (excited at 350 nm. $c = 2.8 \times 10^{-5}$ M).

6. Crystallographic data for 3, 4, 5-sp^3 and 6-sp^3

Table S2. Crystallographic data for **3**, **4**, **5-sp^3** and **6-sp^3**.

	**3**	**4**	**5-sp^3**	**6-sp^3·PhCl**
CCDC	2371388	2371389	2371390	2371391
formula	$C_{23}H_{23}B$	$C_{29}H_{35}B$	$C_{28}H_{20}BBr$	$C_{48}H_{37}B_2Cl$
fw	310.22	394.38	447.16	670.84
crystal dimension	0.28 × 0.24 × 0.08	0.16 × 0.11 × 0.10	0.18 × 0.14 × 0.12	0.16 × 0.15 × 0.05
crystal system	triclinic	monoclinic	monoclinic	triclinic
space group	*P*-1	$P2_1/n$	*C*2/*c*	*P*-1
a [Å]	8.5125(7)	9.6295(9)	18.8181(15)	9.709(11)
b [Å]	9.3289(8)	32.893(2)	22.0129(17)	11.950(12)
c [Å]	22.2417(16)	15.3399(9)	11.7374(11)	16.033(16)
α [deg]	86.859(3)	90	90	105.771(16)
β [deg]	87.797(3)	96.692(2)	119.654(3)	90.871(16)
γ [deg]	87.118(3)	90	90	90.891(14)
V [Å^3]	1760.3(2)	4825.8(6)	4225.3(6)	1789(3)
Z	4	8	8	2
ρ_{calcd} [g cm^{-3}]	1.171	1.086	1.406	1.245
F(000)	664	1712	1824	704
μ [cm^{-1}]	0.65	0.6	19.58	1.42
transmission factors range	0.7142 – 0.7463	0.6711 – 0.7458	0.6914 – 0.7463	0.7034 – 0.7453
index range	−10 ≤ h ≤ 10	−12 ≤ h ≤ 12	−23 ≤ h ≤ 23	−10 ≤ h ≤ 12
	−11 ≤ k ≤ 11	−42 ≤ k ≤ 44	−27 ≤ k ≤ 27	−12 ≤ k ≤ 14
	−28 ≤ l ≤ 28	−20 ≤ l ≤ 20	−14 ≤ l ≤ 14	−19 ≤ l ≤ 19
no. reflections	77860	76504	28658	10690
unique (R_{int})	7677 (0.0531)	12471 (0.0926)	4380 (0.0414)	7081 (0.0172)
$I > 2\sigma(I)$	6805	7161	3603	5362
no. parameters	439	694	326	460
R_1 ($I > 2\sigma(I)$)^a	0.048	0.0615	0.0662	0.0617
wR_2 (all data)^b	0.1296	0.1719	0.1934	0.1868
GOF^c	1.029	1.022	1.037	1.018
max diff peak / hole [e Å^{-3}]	0.32/−0.265	0.259/−0.27	1.774/−1.07	1/−0.757

$^aR_1 = \Sigma||F_o| - |F_c||/\Sigma|F_o|$. $^bwR_2 = [\Sigma\{w(F_o^2 - F_c^2)^2\}f/\Sigma w(F_o^2)^2]^{1/2}$, w = $1/[\sigma^2F_o^2 + (aP)^2 + bP]$ (*a* and *b* are constants suggested by the refinement program; P = $[\max(F_o^2,0) + 2F_c^2]/3$). ^{c}GOF = $[\Sigma w(F_o^2 - F_c^2)^2/(N_{obs} - N_{params})]^{1/2}$.

77. Cartesian coordinates for 1-sp^3, 1-sp^2 and 6-sp^3

1-sp^3:

C	-1.6356440	-2.1052140	-3.0028890
C	-0.9333400	-1.4345920	-2.0361060
C	-1.5958080	-0.7051820	-0.9946940
C	-3.0421990	-0.7001630	-0.9987400
C	-3.7387970	-1.4119330	-2.0254590
C	-3.0602530	-2.0939680	-2.9995640
C	-0.8748250	-0.0000040	-0.0000140
C	-3.7259250	0.0000160	0.0000420
C	-3.0421490	0.7001800	0.9988000
C	-1.5957570	0.7051810	0.9946990
C	-0.9332480	1.4345760	2.0360950
H	0.1532490	1.4561680	2.0506300
C	-1.6355100	2.1051970	3.0029080
C	-3.0601230	2.0939610	2.9996410
C	-3.7387060	1.4119420	2.0255550
H	-1.1066070	-2.6515560	-3.7791470
H	0.1531570	-1.4561720	-2.0506800
H	-4.8264140	-1.3976210	-2.0149010
H	-3.6033290	-2.6296500	-3.7736140
H	-4.8143560	0.0000150	0.0000660
H	-1.1064410	2.6515370	3.7791450
H	-3.6031610	2.6296420	3.7737190
H	-4.8263230	1.3976350	2.0150290
B	0.7218880	-0.0000280	-0.0000210
C	1.4191760	-1.2632780	0.6239250
C	2.7907220	-1.6147510	0.4779470
C	0.6043890	-2.1600080	1.3566430
C	3.2669700	-2.8090950	1.0296520
C	1.0913350	-3.3354560	1.9205300
H	-0.4457430	-1.9202250	1.4856380
C	2.4334510	-3.6671880	1.7469920
H	4.3160940	-3.0674490	0.9037870
H	0.4284830	-3.9879630	2.4823780
H	2.8352160	-4.5840770	2.1714440
C	1.4191500	1.2632530	-0.6239740
C	0.6043310	2.1599890	-1.3566430

C	2.7906910	1.6147490	-0.4780240
C	1.0912500	3.3354510	-1.9205260
H	-0.4458060	1.9202140	-1.4856070
C	3.2669140	2.8091090	-1.0297150
C	2.4333670	3.6672000	-1.7470240
H	0.4283690	3.9879670	-2.4823310
H	4.3160350	3.0674770	-0.9038540
H	2.8351040	4.5841070	-2.1714650
C	3.7806150	-0.7297890	-0.2475500
H	3.5863230	-0.7384710	-1.3293980
H	4.7832040	-1.1520650	-0.1151930
C	3.7805960	0.7298180	0.2474910
H	3.5862870	0.7385010	1.3293350
H	4.7831770	1.1521170	0.1151430

1-sp^2:

C	1.5661480	-0.0006910	-3.6590940
C	0.8700650	-0.0004700	-2.4796810
C	1.5429060	-0.0002300	-1.2164020
C	2.9874000	-0.0002300	-1.2189530
C	3.6769980	-0.0004660	-2.4705050
C	2.9907710	-0.0006900	-3.6551350
C	0.8275020	-0.0000010	0.0000310
C	3.6717390	-0.0000010	0.0001110
C	2.9873310	0.0002280	1.2191370
C	1.5428370	0.0002280	1.2165050
C	0.8699250	0.0004680	2.4797460
H	-0.2165860	0.0004710	2.4909920
C	1.5659410	0.0006890	3.6591970
C	2.9905640	0.0006880	3.6553190
C	3.6768580	0.0004640	2.4707280
H	1.0340110	-0.0008710	-4.6061210
H	-0.2164450	-0.0004720	-2.4909890
H	4.7643050	-0.0004640	-2.4599090
H	3.5288240	-0.0008670	-4.5989540
H	4.7596990	-0.0000010	0.0001420
H	1.0337510	0.0008690	4.6061940
H	3.5285640	0.0008650	4.5991690

H	4.7641660	0.0004620	2.4601930
B	-0.7624040	0.0000000	-0.0000150
C	-1.4412360	1.3959870	-0.0002510
C	-2.8443740	1.6656640	-0.0003440
C	-0.5774580	2.5204140	-0.0003800
C	-3.2921860	3.0080980	-0.0005570
C	-1.0337370	3.8291740	-0.0005880
H	0.4917240	2.3457460	-0.0003130
C	-2.4111230	4.0750160	-0.0006770
H	-4.3622780	3.1982360	-0.0006260
H	-0.3271640	4.6541220	-0.0006800
H	-2.7899380	5.0935040	-0.0008390
C	-1.4412380	-1.3959860	0.0001690
C	-0.5774620	-2.5204140	0.0003710
C	-2.8443760	-1.6656610	0.0001500
C	-1.0337420	-3.8291740	0.0005470
H	0.4917210	-2.3457480	0.0003880
C	-3.2921900	-3.0080940	0.0003310
C	-2.4111290	-4.0750130	0.0005270
H	-0.3271700	-4.6541230	0.0006990
H	-4.3622820	-3.1982310	0.0003150
H	-2.7899450	-5.0935010	0.0006620
C	-3.9003330	0.6776670	-0.0002420
H	-4.8946700	1.1215990	-0.0003490
C	-3.9003340	-0.6776630	-0.0000410
H	-4.8946710	-1.1215930	-0.0000180

6-sp^3:

C	-0.7112010	-0.0075680	3.6655750
C	-1.3979860	-0.0127550	2.4808420
C	-0.7237130	-0.0067740	1.2151170
C	0.7238660	0.0067420	1.2150220
C	1.3982990	0.0126210	2.4806650
C	0.7116630	0.0073360	3.6654880
C	-1.4480660	0.0000430	0.0002180
C	1.4480570	0.0000150	0.0000210
C	0.7237020	-0.0066970	-1.2148810
C	-0.7238760	0.0068470	-1.2147780

C	-1.3983230	0.0128410	-2.4804100
H	-2.4851350	0.0280720	-2.4911880
C	-0.7117000	0.0076420	-3.6652380
C	0.7111630	-0.0072900	-3.6653450
C	1.3979620	-0.0125890	-2.4806170
H	-1.2498810	-0.0151460	4.6094360
H	-2.4847960	-0.0279640	2.4917690
H	2.4851080	0.0278370	2.4914570
H	1.2504570	0.0148320	4.6092840
H	-1.2505100	0.0152310	-4.6090250
H	1.2498280	-0.0147960	-4.6092150
H	2.4847690	-0.0278260	-2.4915580
B	-3.0440760	0.0000570	0.0003250
C	-3.7435260	-1.3931540	0.2101320
C	-5.1147510	-1.5976870	0.5317900
C	-2.9297050	-2.5481020	0.1203600
C	-5.5915070	-2.8928520	0.7624140
C	-3.4169410	-3.8347610	0.3299790
H	-1.8799490	-2.4244200	-0.1238800
C	-4.7587040	-4.0073640	0.6636230
H	-6.6405190	-3.0323100	1.0142550
H	-2.7547580	-4.6917980	0.2409030
H	-5.1607030	-5.0021620	0.8399060
C	-3.7435370	1.3932130	-0.2098270
C	-2.9298120	2.5482140	-0.1198640
C	-5.1146730	1.5976490	-0.5319380
C	-3.4170560	3.8348330	-0.3297060
H	-1.8801280	2.4246090	0.1247230
C	-5.5914300	2.8927750	-0.7627780
C	-4.7587240	4.0073410	-0.6637820
H	-2.7549520	4.6919140	-0.2404660
H	-6.6403680	3.0321590	-1.0149660
H	-5.1607250	5.0021060	-0.8402460
C	-6.1044660	-0.4566330	0.6205950
H	-5.9099100	0.1556420	1.5124830
H	-7.1071130	-0.8785230	0.7539630
C	-6.1042950	0.4565360	-0.6210370
H	-5.9094230	-0.1557340	-1.5128600

H	-7.1069240	0.8783670	-0.7547270
B	3.0440830	-0.0000010	-0.0000870
C	3.7435710	1.3931780	0.2098760
C	3.7435110	-1.3932080	-0.2100700
C	5.1147610	1.5976200	0.5317560
C	2.9298180	2.5481800	0.1201080
C	2.9297240	-2.5481810	-0.1202340
C	5.1146720	-1.5977000	-0.5320340
C	5.5915450	2.8927400	0.7625640
C	6.1044150	0.4565230	0.6206370
C	3.4170920	3.8347980	0.3299040
H	1.8800920	2.4245800	-0.1242820
C	3.4169420	-3.8348180	-0.3300420
H	1.8800170	-2.4245420	0.1242200
C	5.5913990	-2.8928390	-0.7628540
C	6.1043570	-0.4566350	-0.6209870
C	4.7588150	4.0073020	0.6637520
H	6.6405280	3.0321290	1.0145680
H	5.9097550	-0.1557500	1.5125060
H	7.1070650	0.8783770	0.7541040
H	2.7549700	4.6918790	0.2408110
C	4.7586380	-4.0073710	-0.6639730
H	2.7547970	-4.6918760	-0.2408960
H	6.6403620	-3.0322660	-1.0149220
H	5.9096560	0.1556430	-1.5128430
H	7.1069850	-0.8785230	-0.7545230
H	5.1608380	5.0020630	0.8401910
H	5.1606170	-5.0021480	-0.8404240

8. NMR spectra of the products

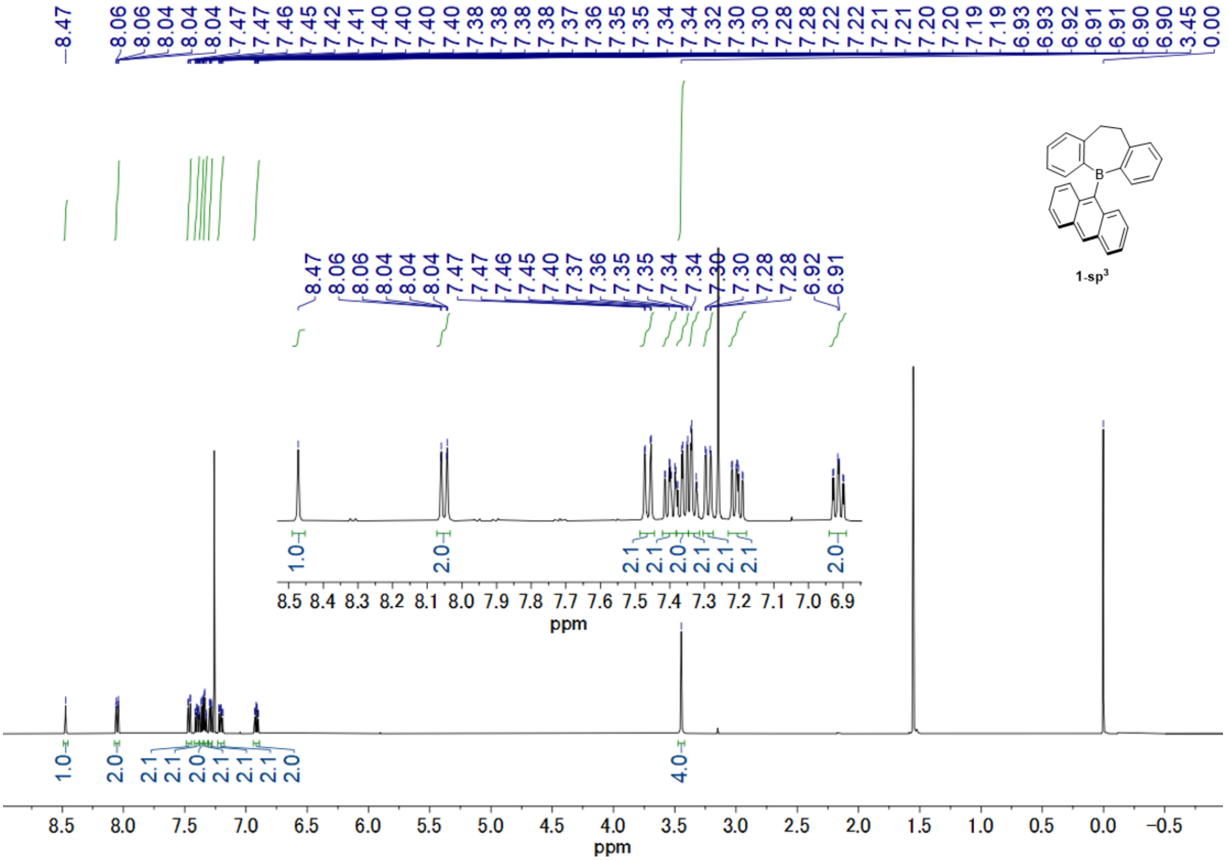

Figure S5. ^{1}H NMR spectrum of **1a** ($CDCl_3$, 500 MHz).

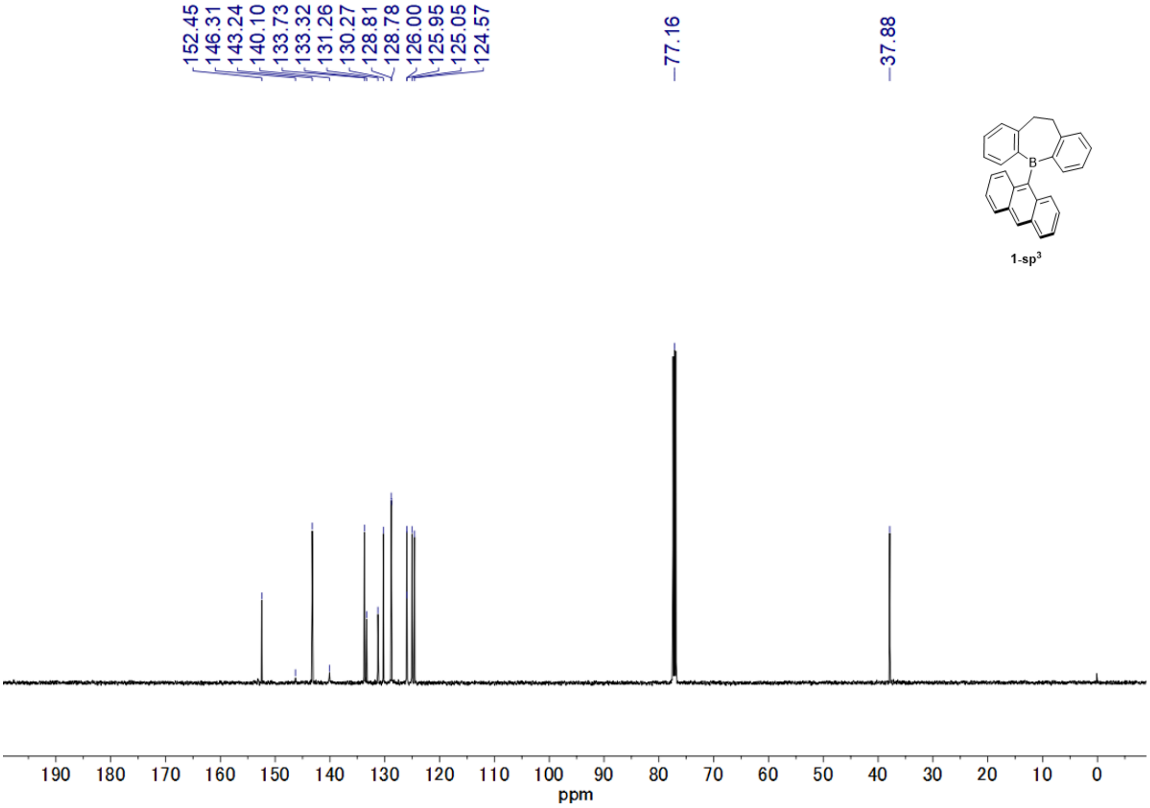

Figure S6. $^{13}C\{^1H\}$ NMR spectrum of **1a** ($CDCl_3$, 126 MHz).

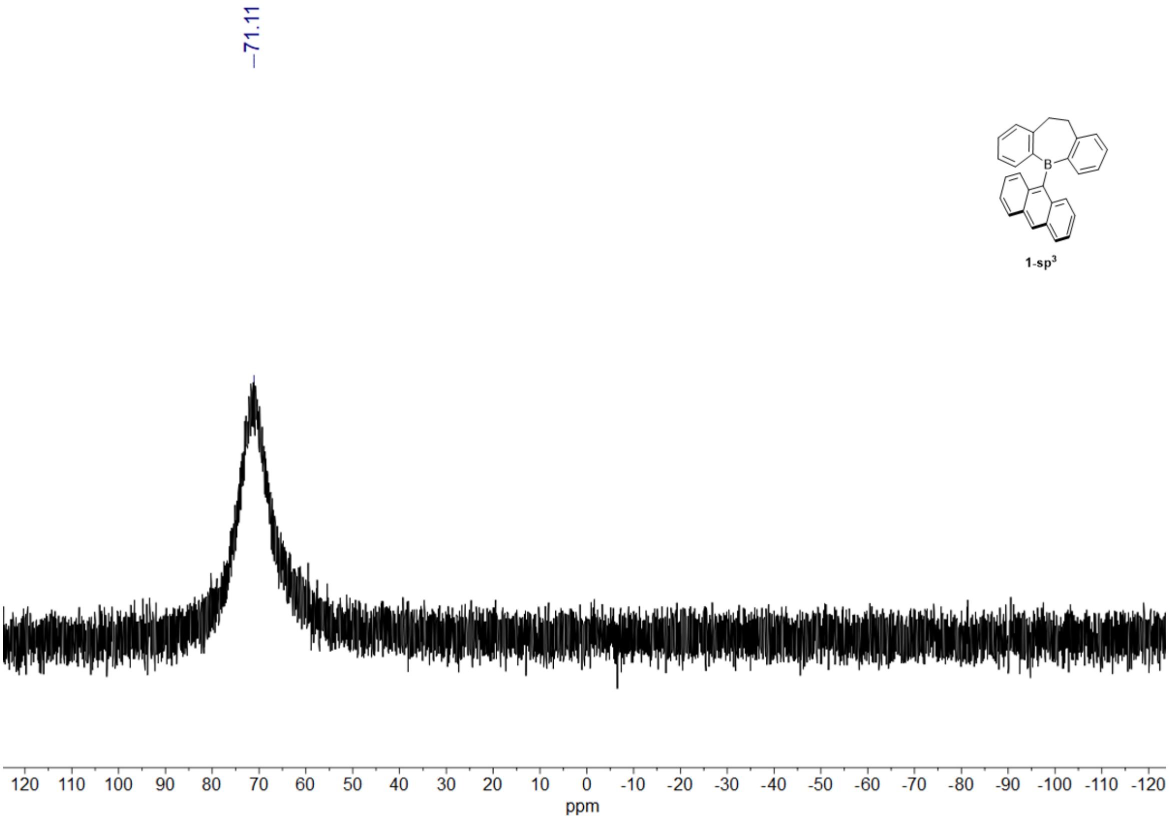

Figure S7. $^{11}B\{^{1}H\}$ NMR spectrum of **1a** ($CDCl_3$, 160 MHz).

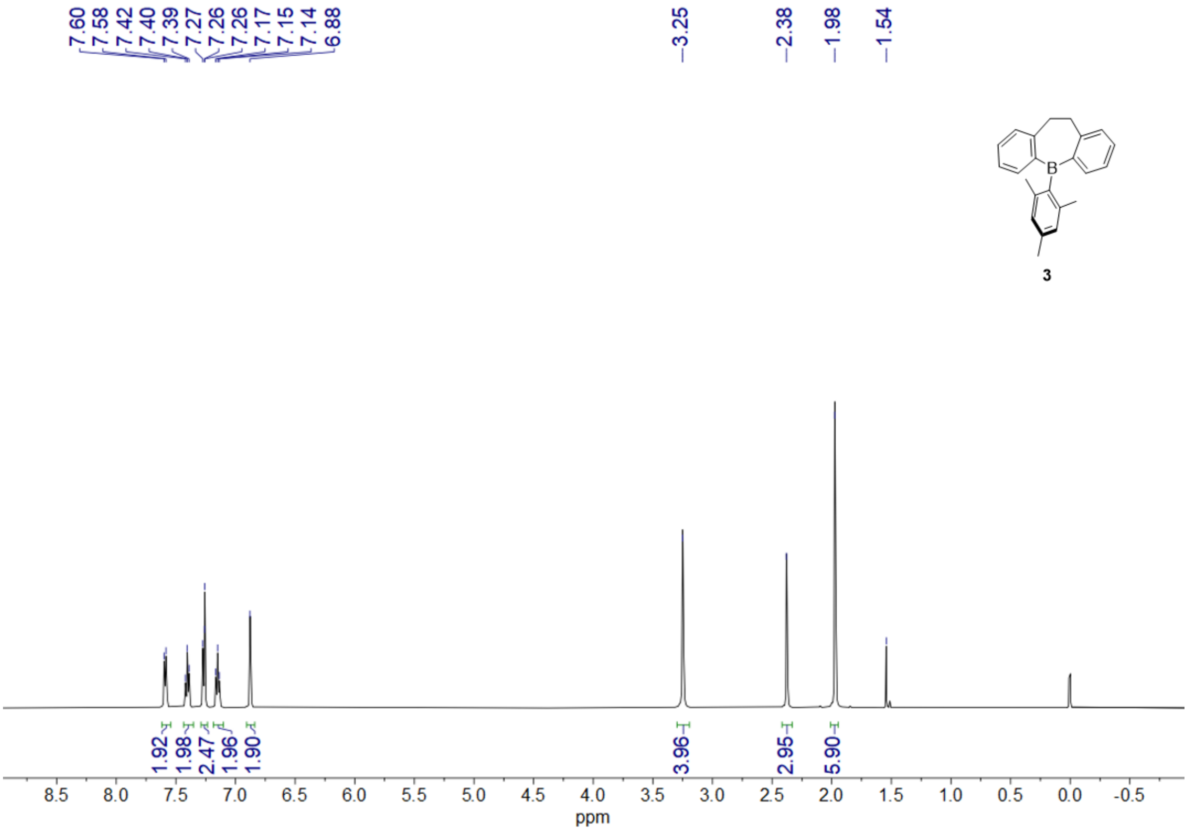

Figure S8. ^{1}H NMR spectrum of **3** ($CDCl_3$, 500 MHz).

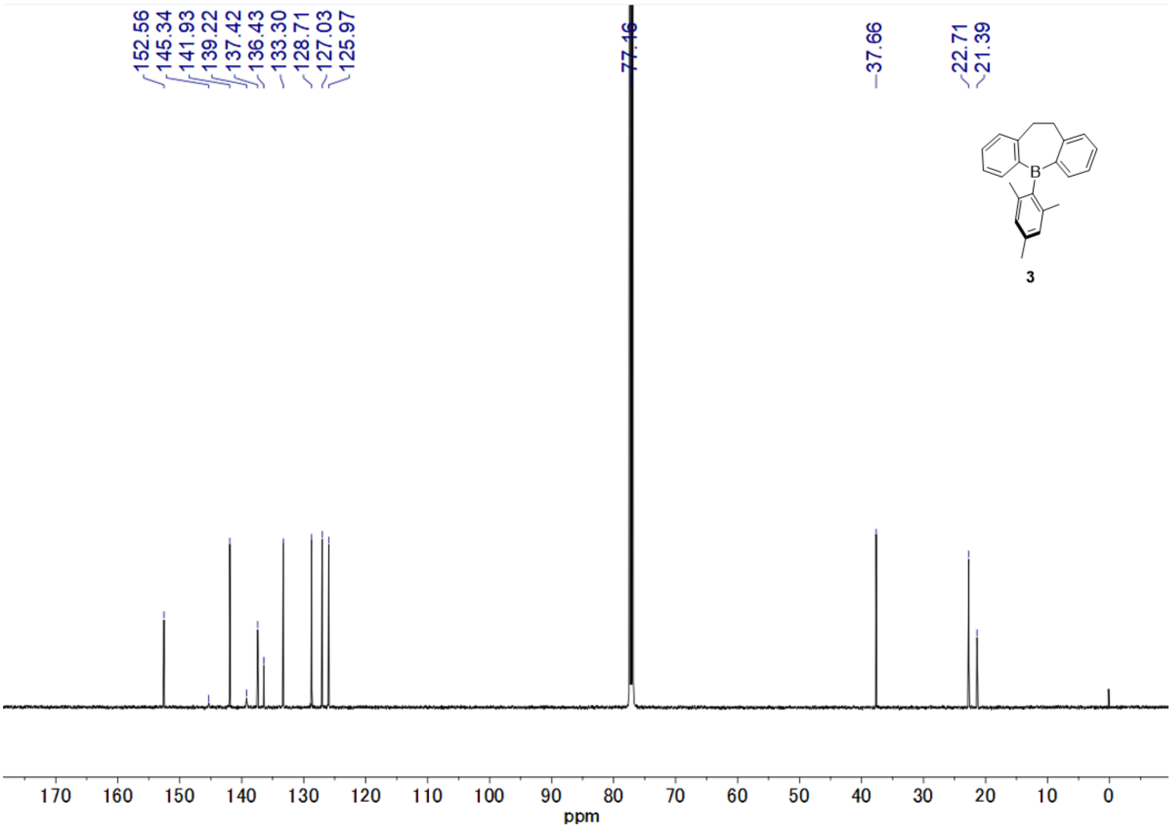

Figure S9. $^{13}C\{^{1}$ NMR spectrum of **3** ($CDCl_3$, 126 MHz).

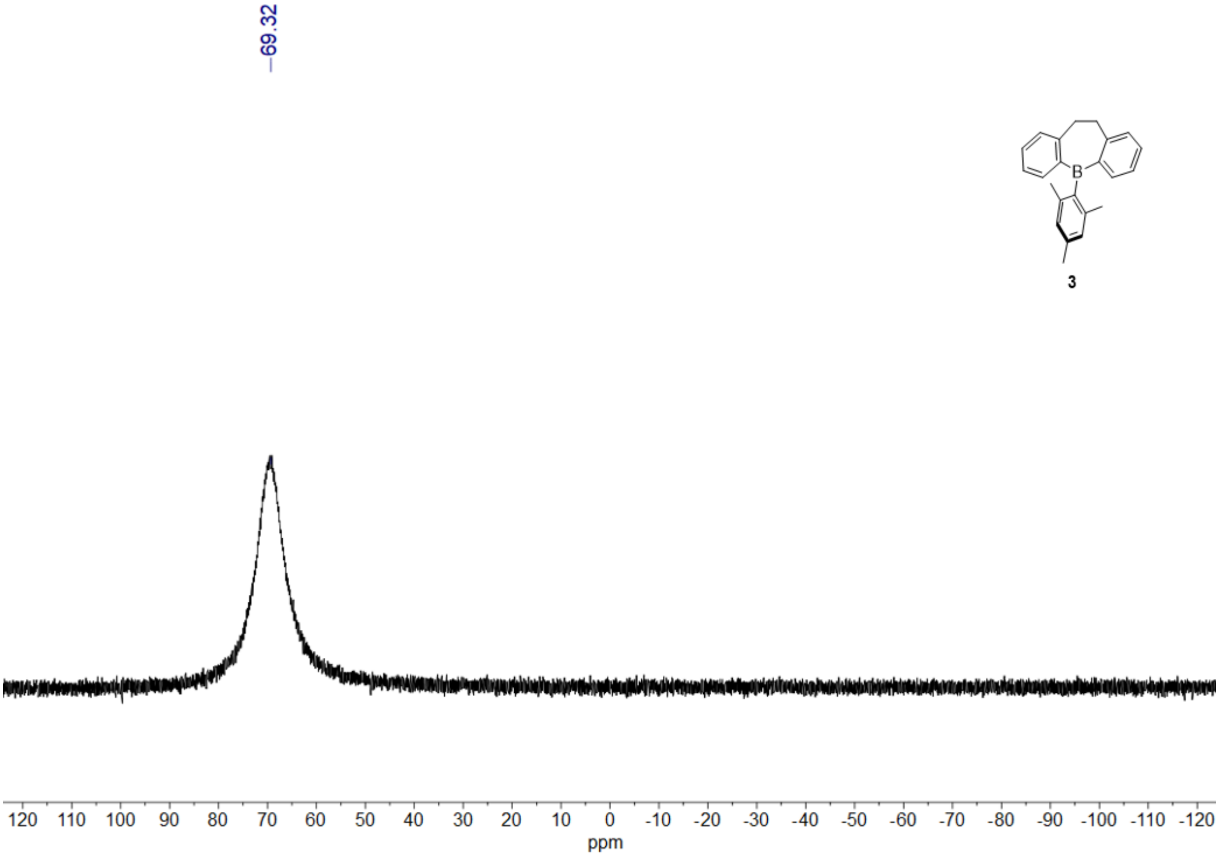

Figure S10. $^{11}B\{^{1}H\}$ NMR spectrum of **3** ($CDCl_3$, 160 MHz).

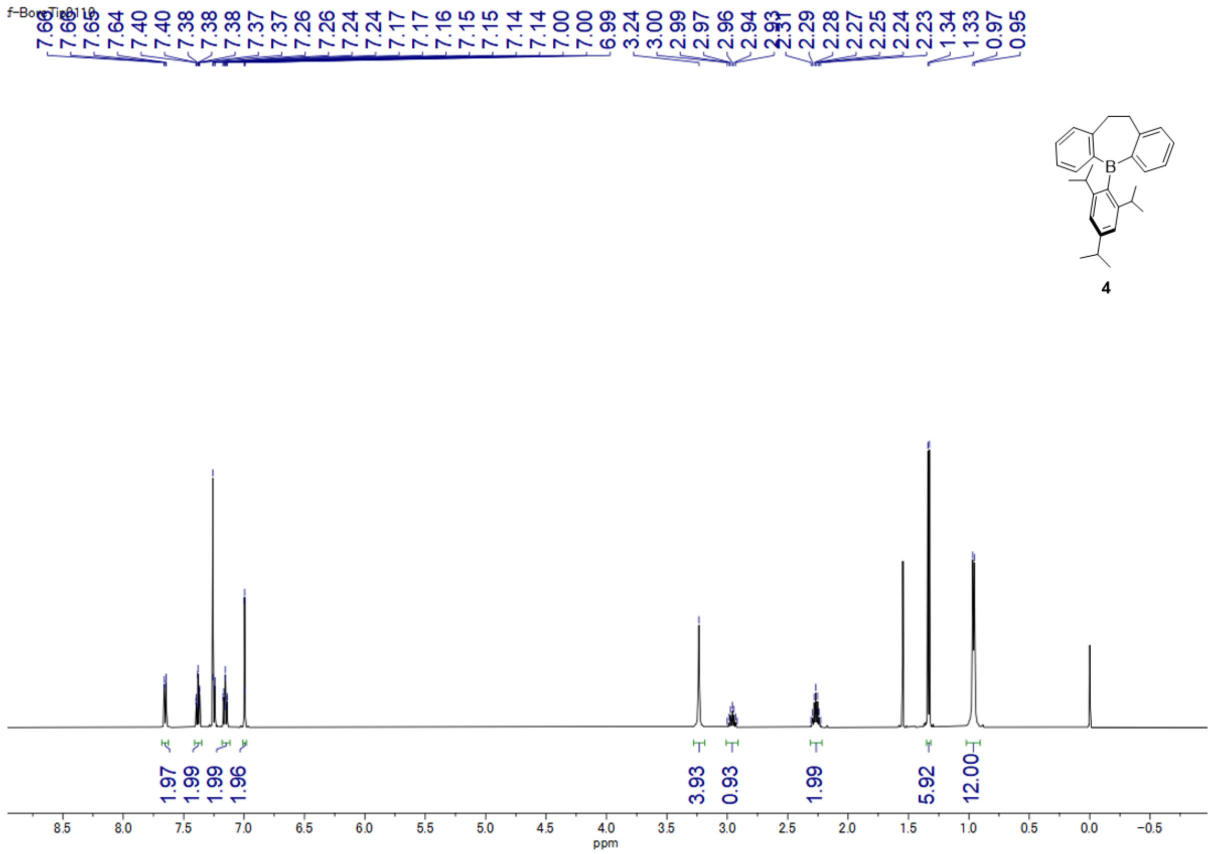

Figure S11. ^{1}H NMR spectrum of **4** ($CDCl_3$, 500 MHz).

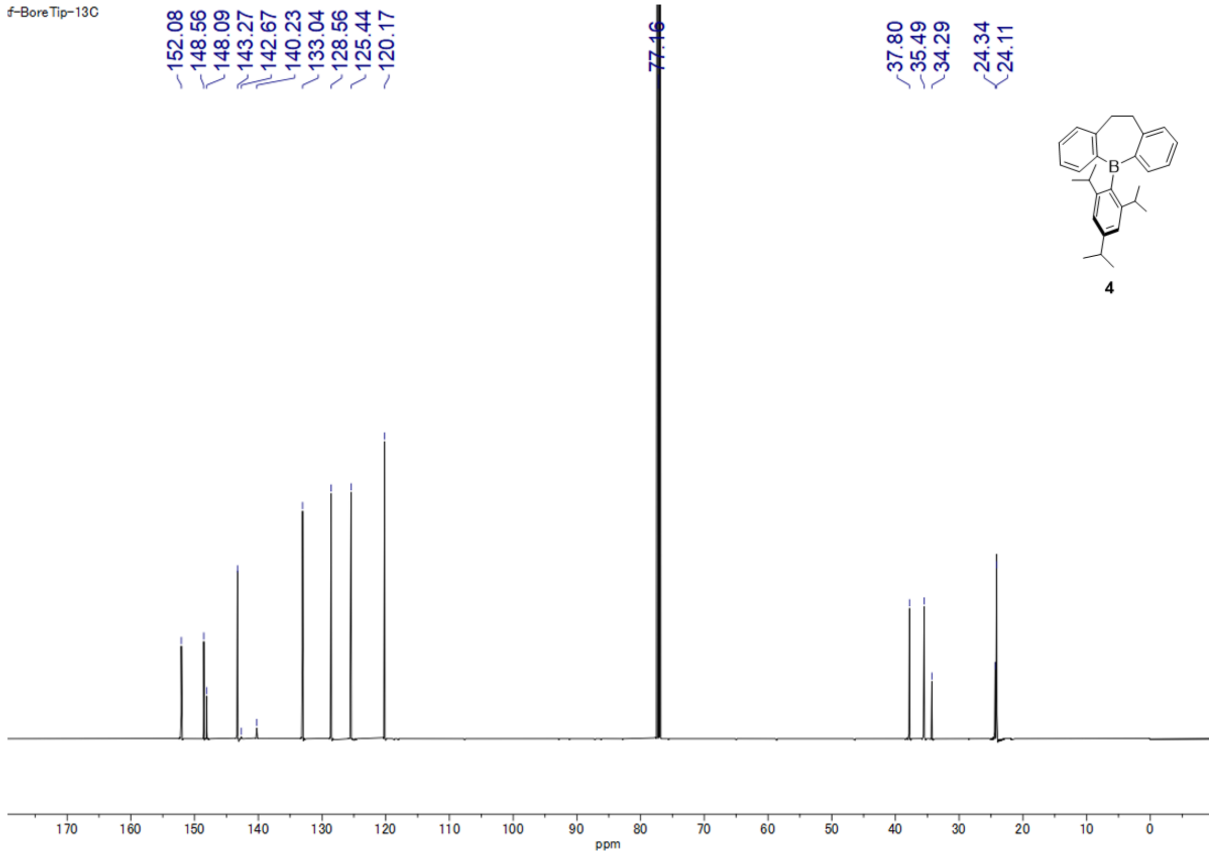

Figure S12. $^{13}C\{^{1}H\}$ NMR spectrum of **4** ($CDCl_3$, 126 MHz).

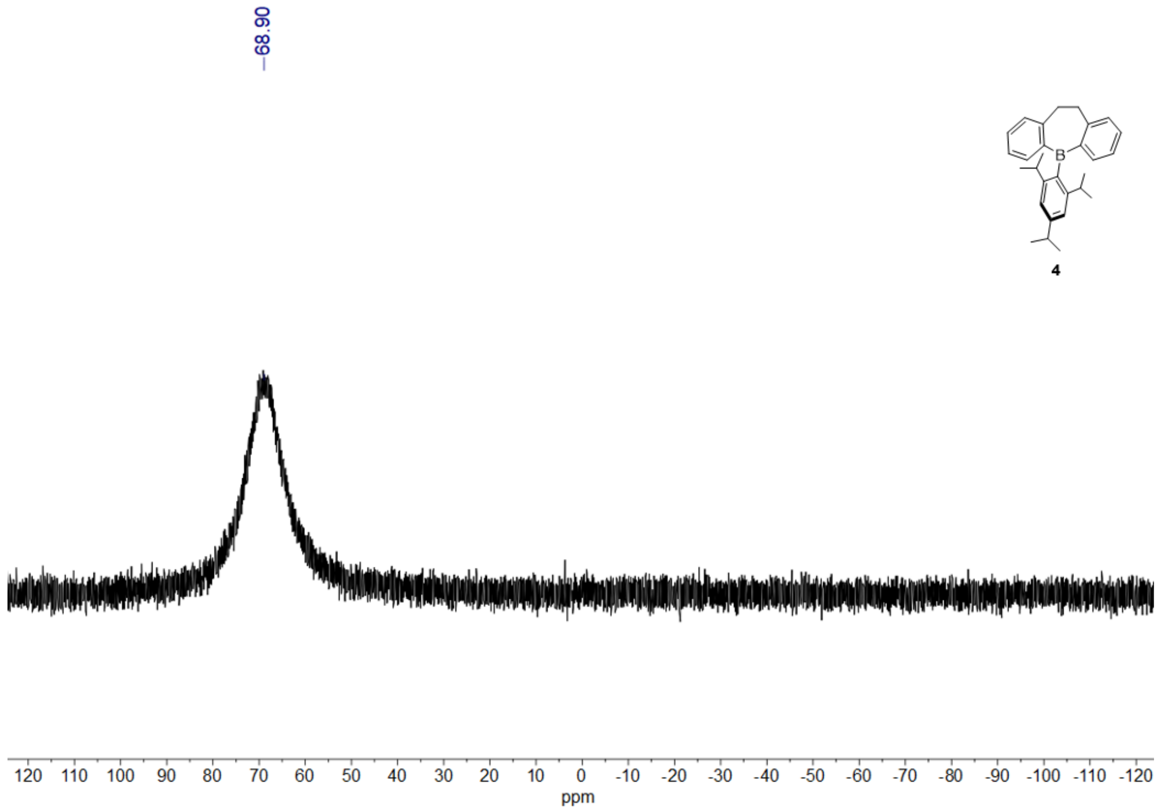

Figure S13. $^{11}B\{^1H\}$ NMR spectrum of **4** ($CDCl_3$, 160 MHz).

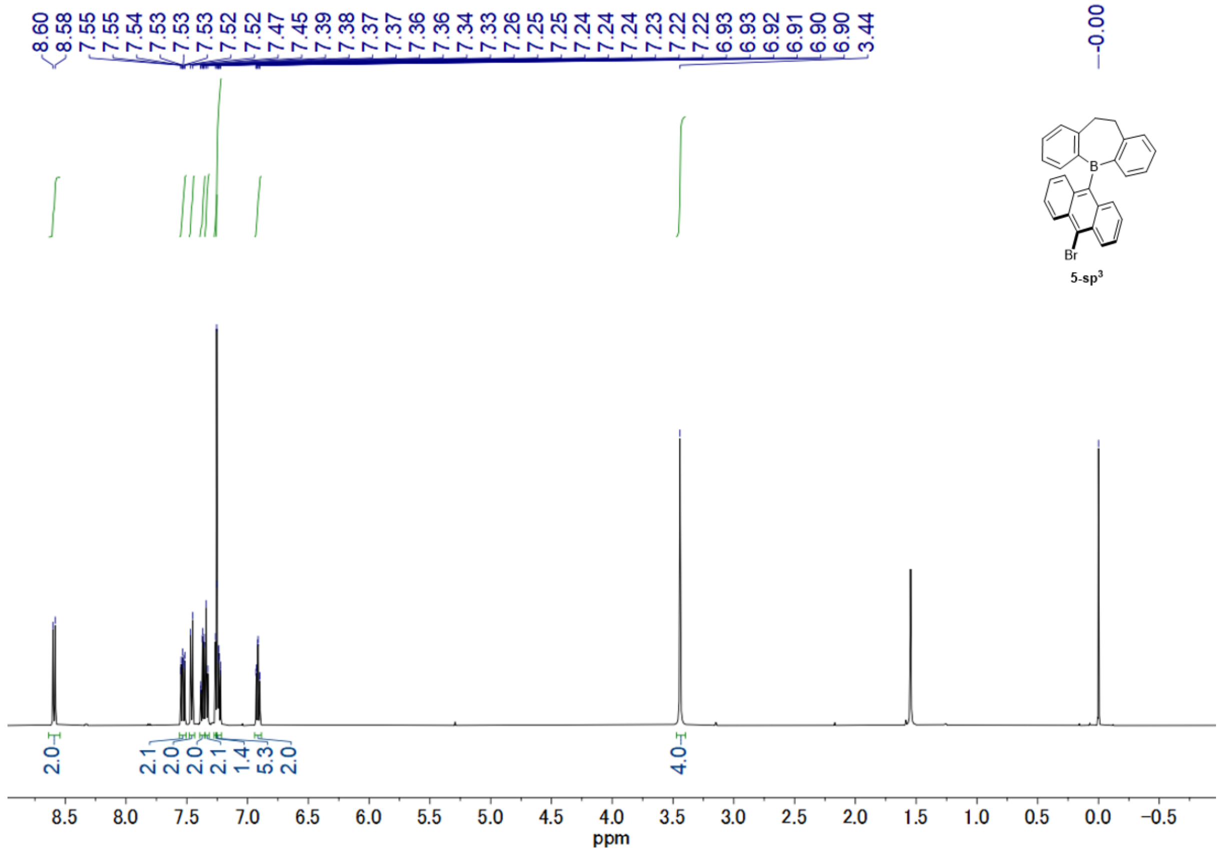

Figure S14. ^1H NMR spectrum of **5-sp^3** ($CDCl_3$, 500 MHz).

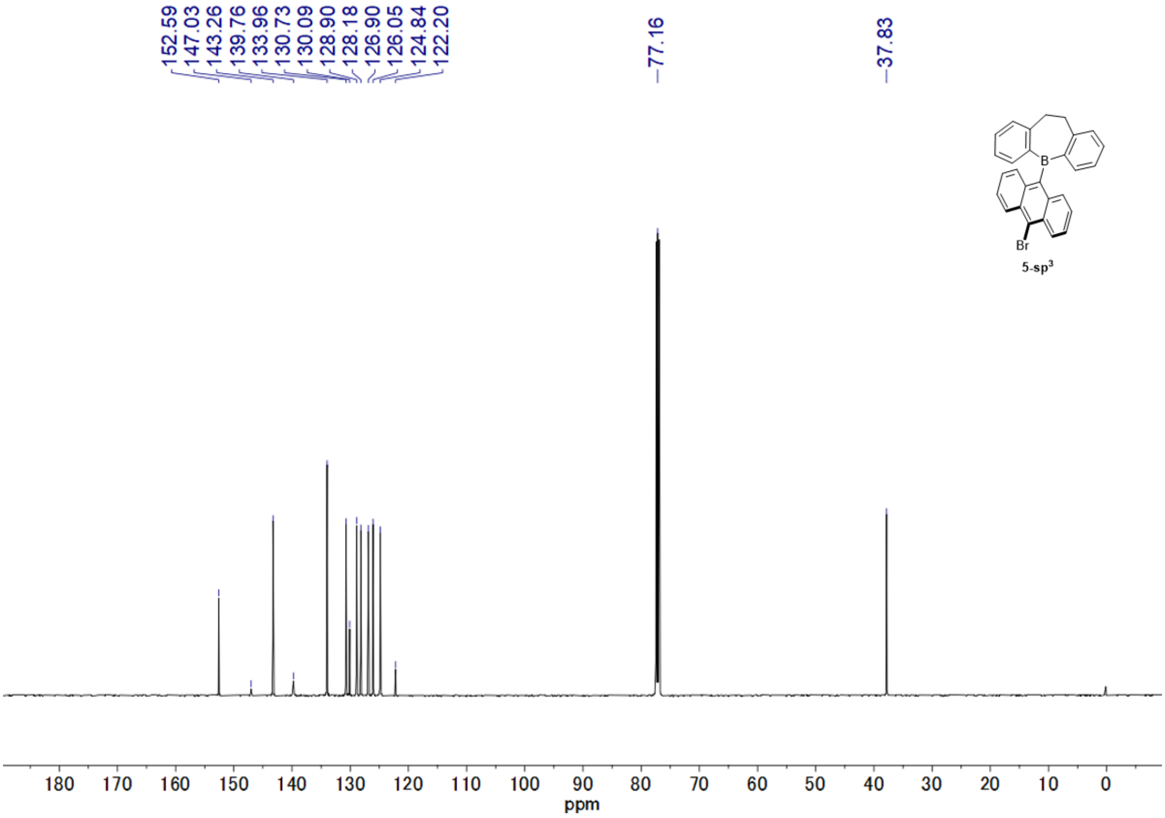

Figure S15. $^{13}C\{^{1}H\}$ NMR spectrum of **5-sp^3** ($CDCl_3$ 126 MHz).

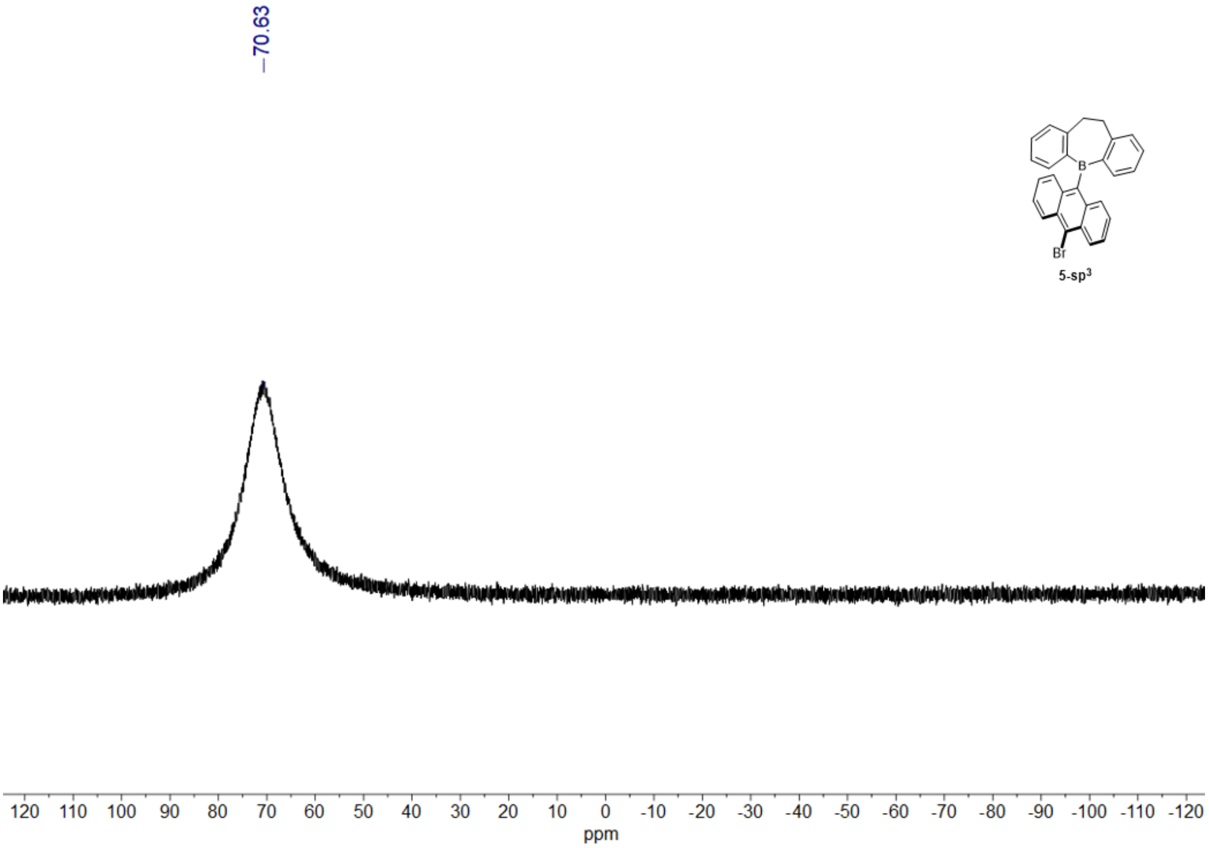

Figure S16. $^{11}B\{^{1}H\}$ NMR spectrum of **5-sp^3** ($CDCl_3$, 160 MHz).

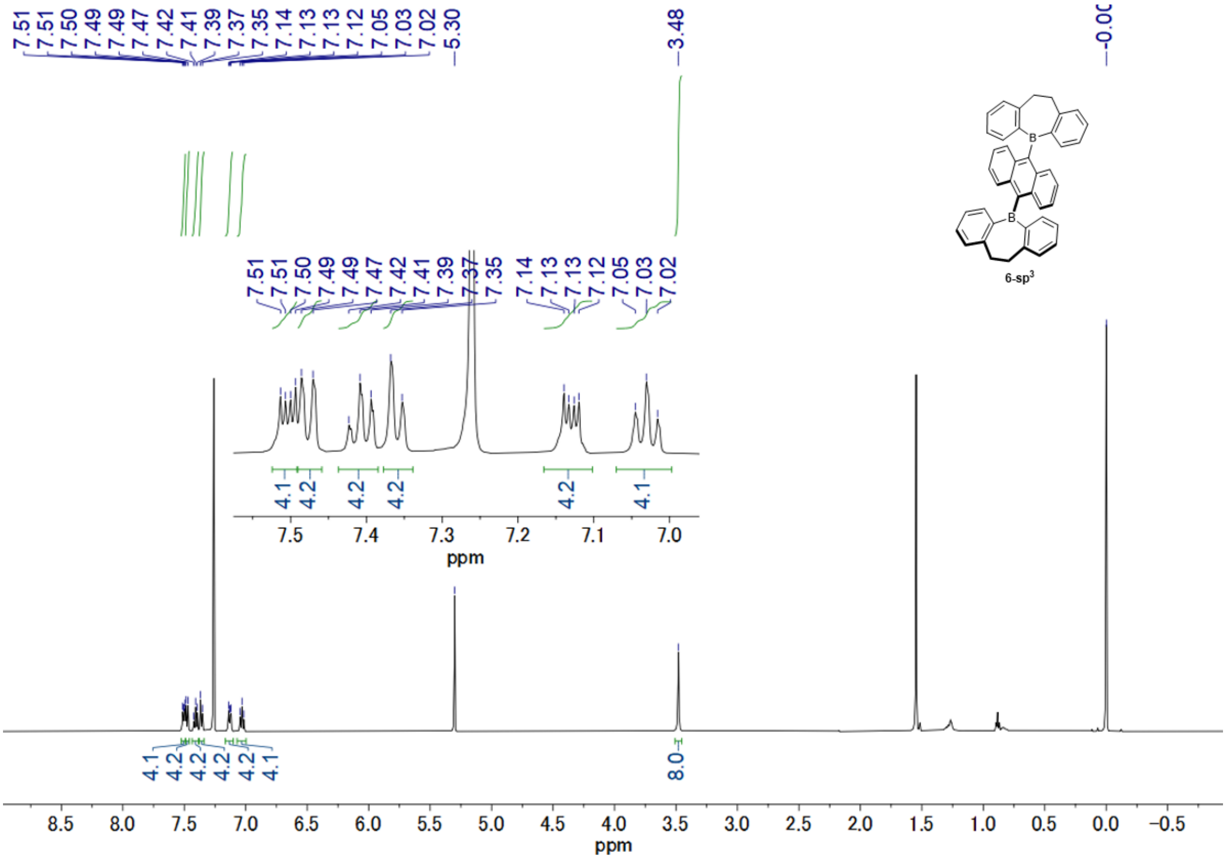

Figure S17. ^{1}H NMR spectrum of **6-sp^3** ($CDCl_3$, 500 MHz).

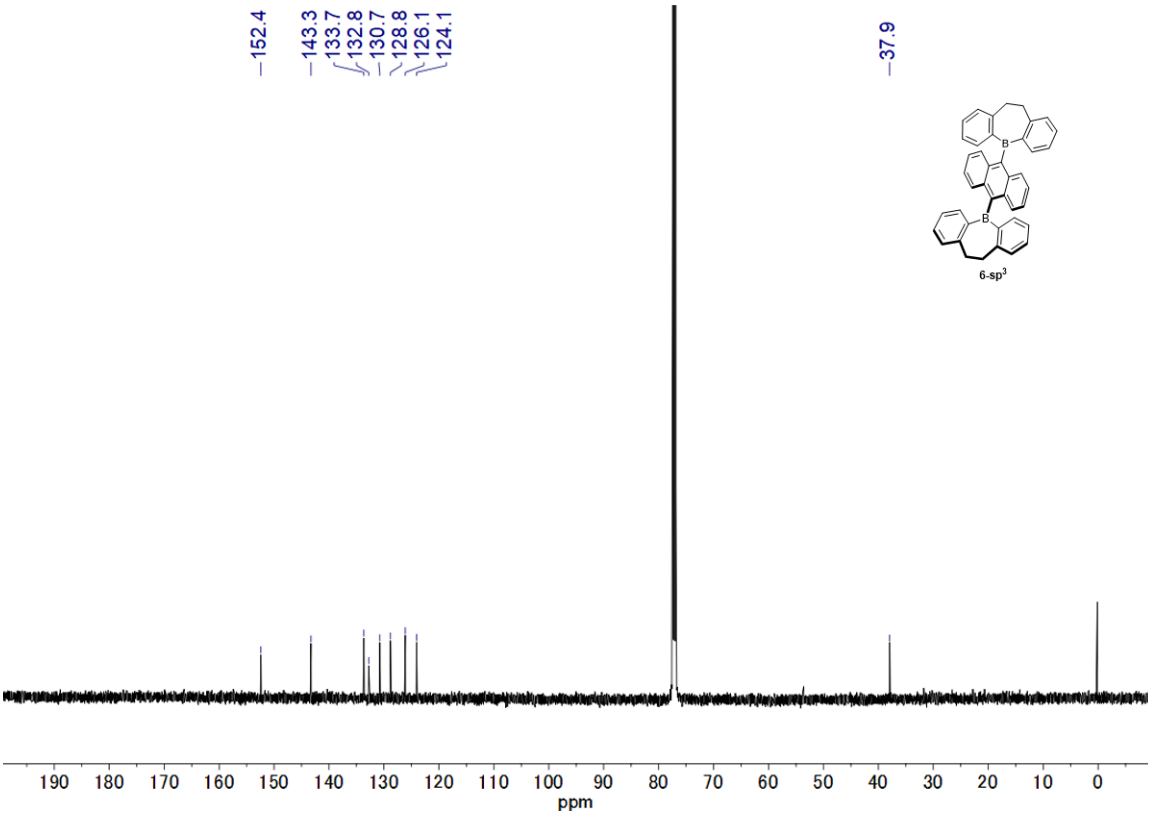

Figure S18. $^{13}C\{^1H\}$ NMR spectrum of **6-sp^3** ($CDCl_3$, 126 MHz).

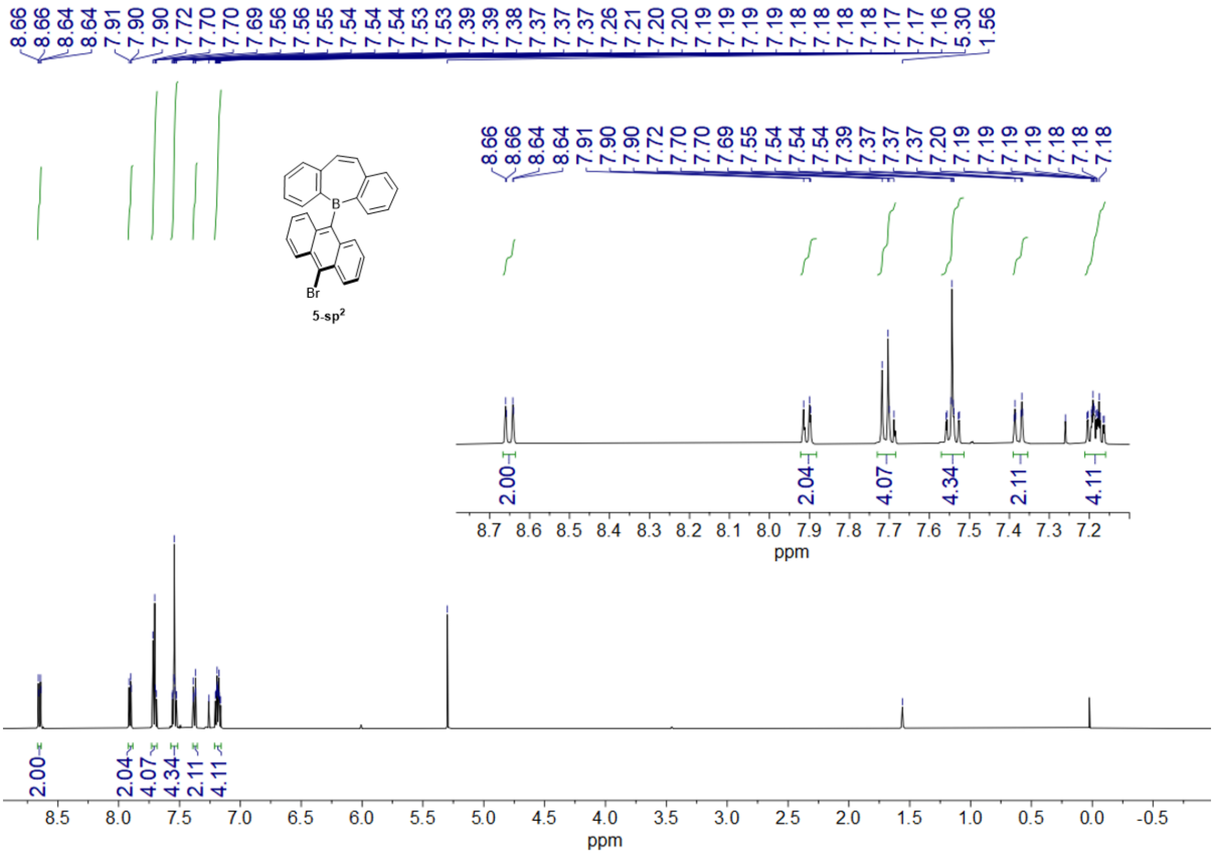

Figure S19. ^{1}H NMR spectrum of **5-sp²** ($CDCl_3$, 500 MHz).

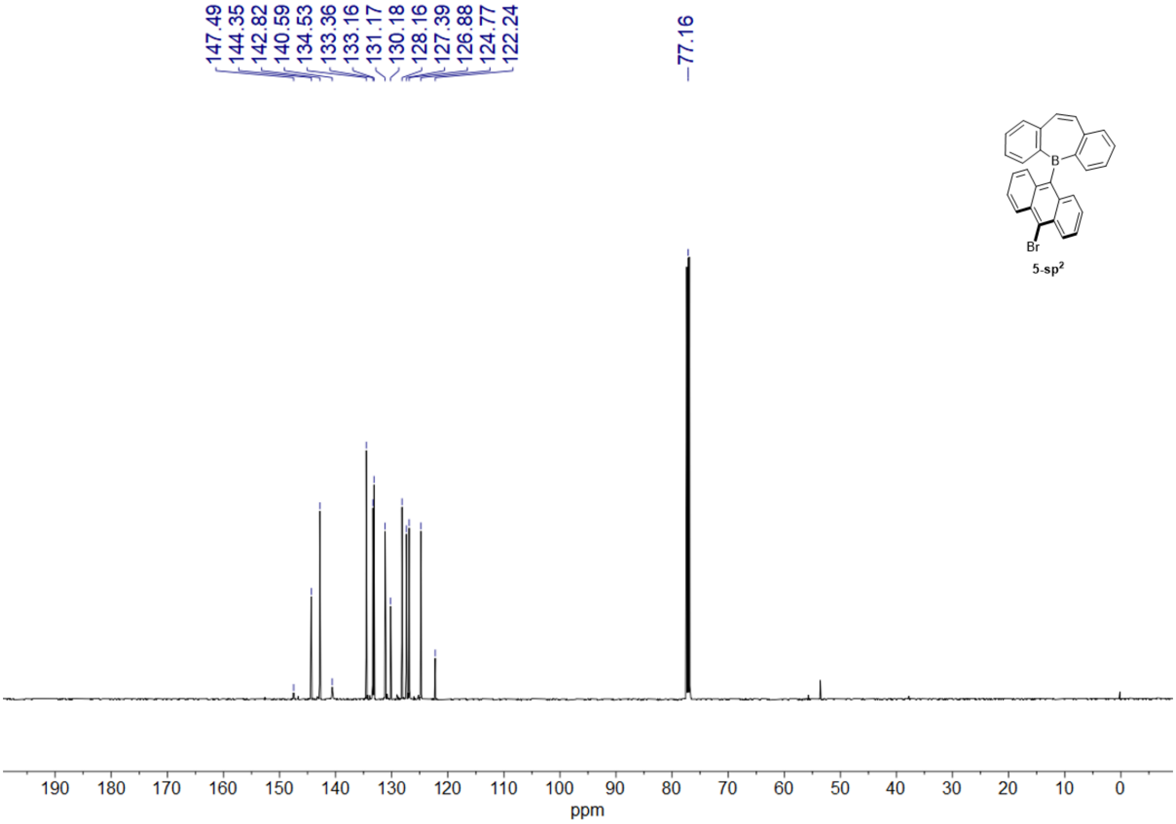

Figure S20. $^{13}C\{^{1}H\}$ NMR spectrum of **5-sp²** ($CDCl_3$, 500 MHz).

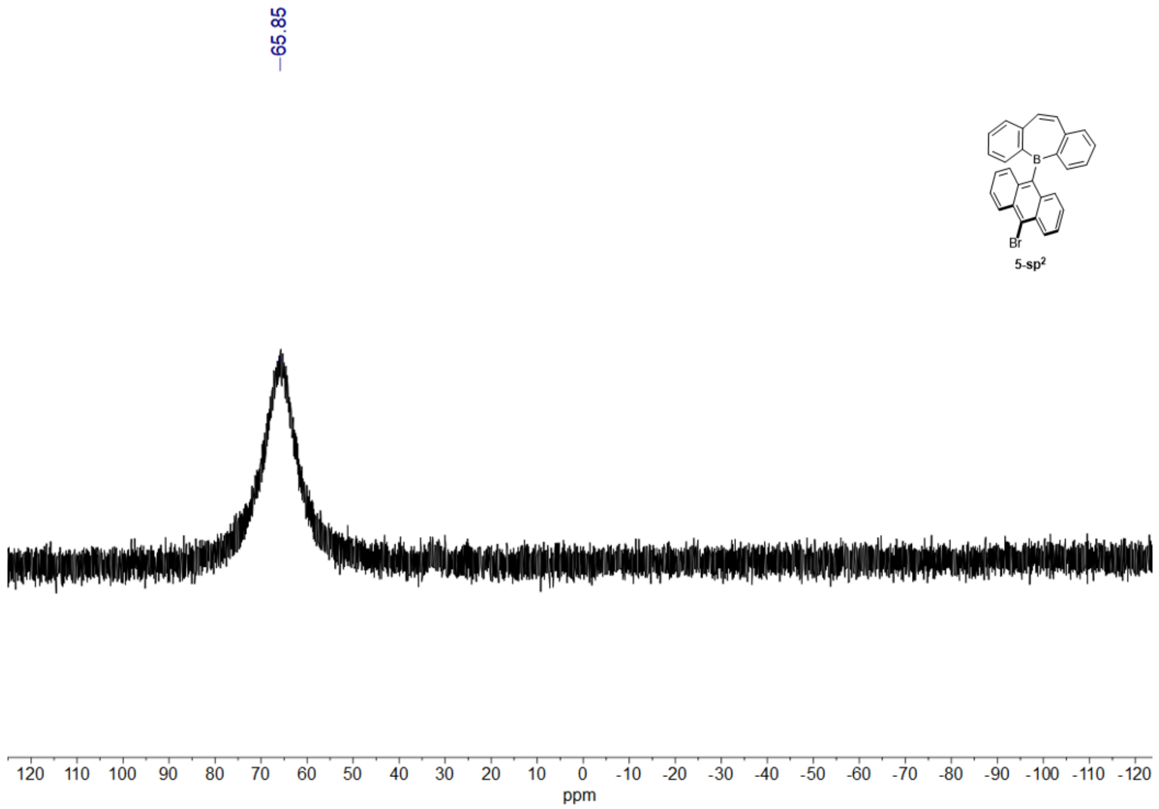

Figure S21. $^{11}B\{^{1}H\}$ NMR spectrum of **5-sp²** ($CDCl_3$, 160 MHz).

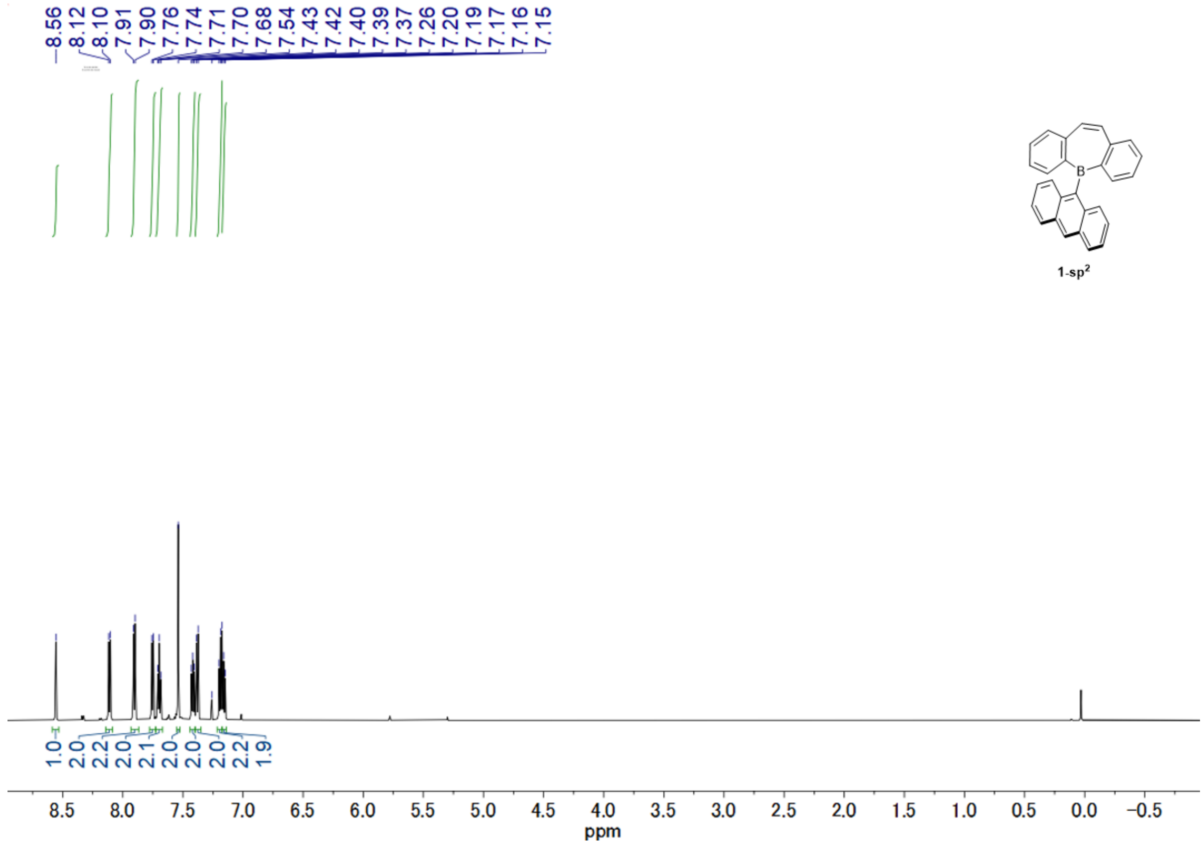

Figure S22. ^{1}H NMR spectrum of **1-sp²** ($CDCl_3$, 500 MHz).

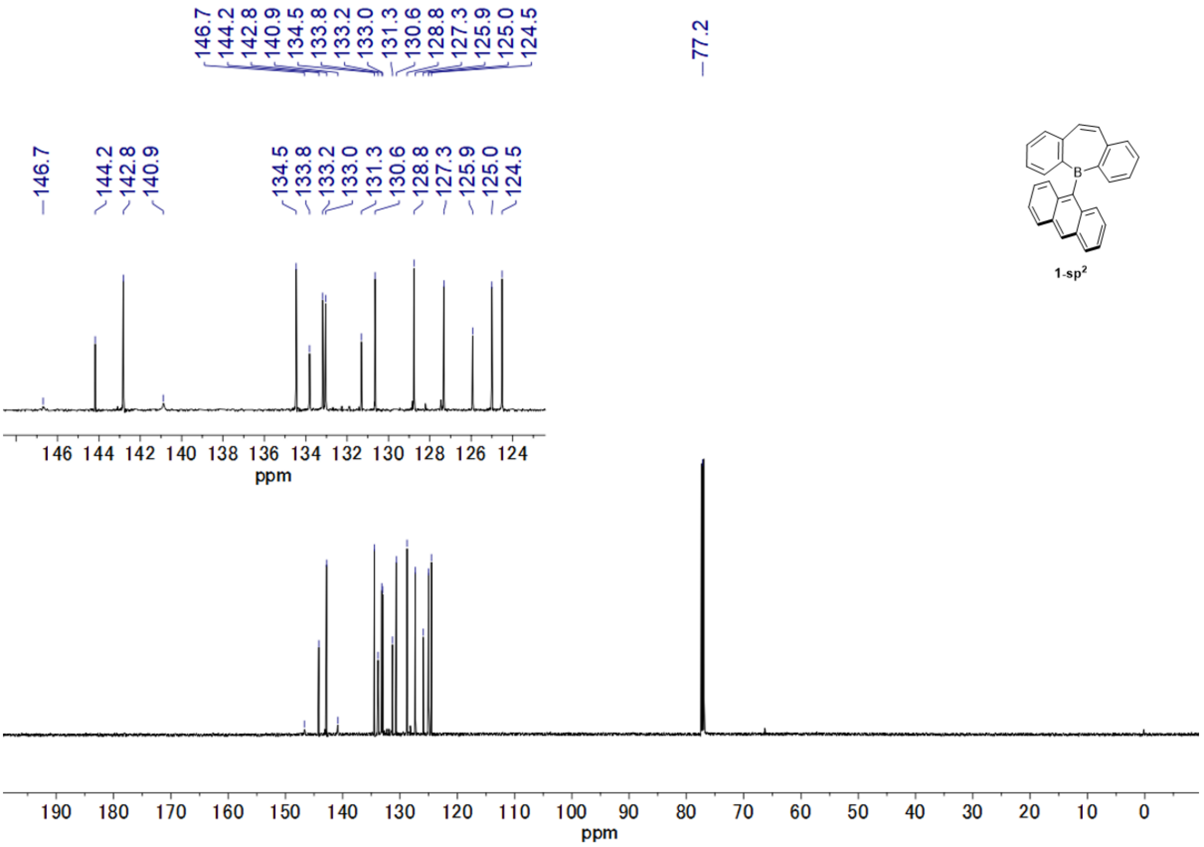

Figure S23. $^{13}C\{^{1}H\}$ NMR spectrum of **1-sp²** ($CDCl_3$, 126 MHz).

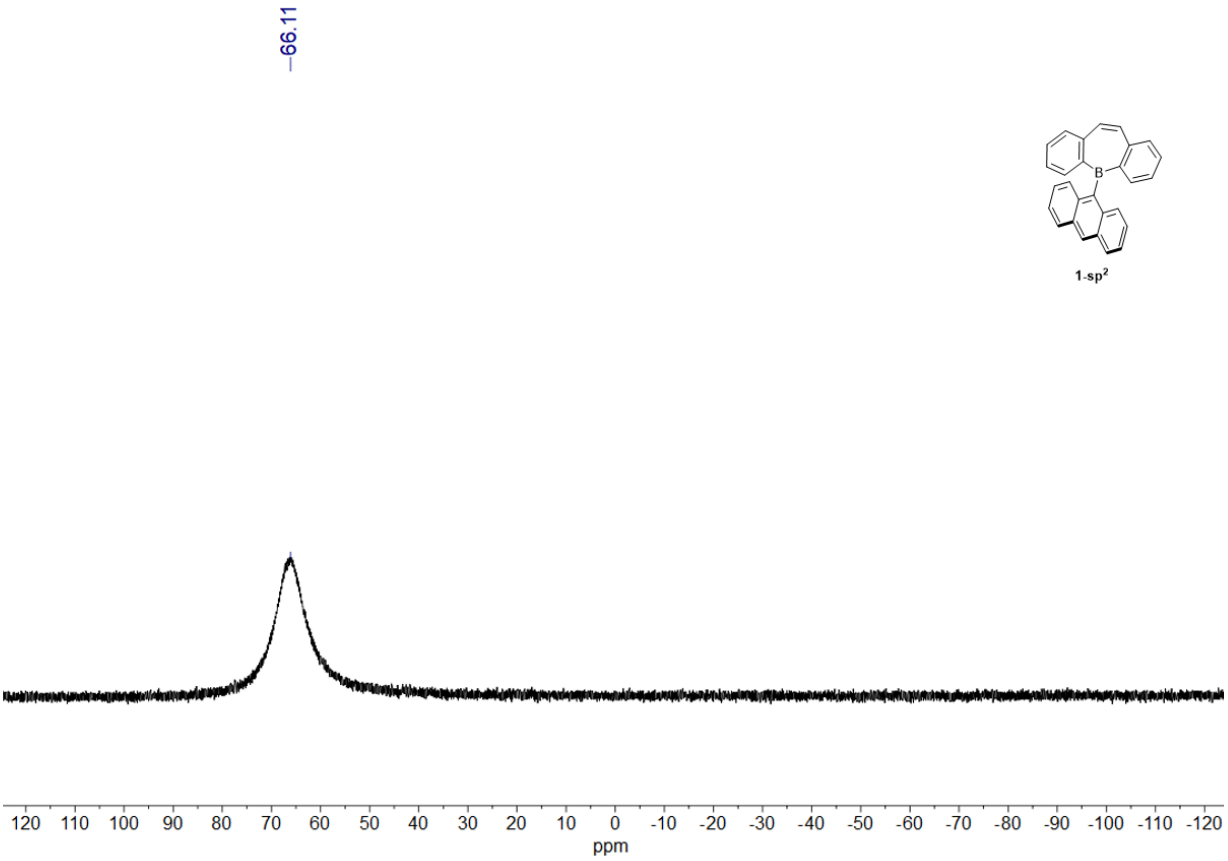

Figure S24. $^{11}B\{^{1}H\}$ NMR spectrum of **1-sp²** ($CDCl_3$, 160 MHz).
